# Multimodular flavobacterial enzymes specialized in coordinated decomposition of cellulose and alginate in brown algal cell walls

**DOI:** 10.1093/ismejo/wrag112

**Published:** 2026-05-08

**Authors:** Fei Xu, Xiao-Hui Sun, Xiao-Dong Zhang, Xiao-Fei Wang, Yan Wang, Hai-Yan Cao, Peng Wang, Jian-Xun Li, Xi-Ying Zhang, Qi-Long Qin, Xiu-Lan Chen, Yu-Zhong Zhang, Yin Chen, Yu-Qiang Zhang

**Affiliations:** State Key Laboratory of Microbial Technology, Shandong University, Qingdao 266237, Shandong Province, China; State Key Laboratory of Microbial Technology, Shandong University, Qingdao 266237, Shandong Province, China; State Key Laboratory of Microbial Technology, Shandong University, Qingdao 266237, Shandong Province, China; State Key Laboratory of Microbial Technology, Shandong University, Qingdao 266237, Shandong Province, China; State Key Laboratory of Microbial Technology, Shandong University, Qingdao 266237, Shandong Province, China; MOE Key Laboratory of Evolution and Marine Biodiversity, Frontiers Science Center for Deep Ocean Multispheres and Earth System & College of Marine Life Sciences, Ocean University of China, Qingdao 266203, Shandong Province, China; MOE Key Laboratory of Evolution and Marine Biodiversity, Frontiers Science Center for Deep Ocean Multispheres and Earth System & College of Marine Life Sciences, Ocean University of China, Qingdao 266203, Shandong Province, China; Laboratory for Marine Biology and Biotechnology, Qingdao Marine Science and Technology Center & Laoshan Laboratory, Qingdao 266237, Shandong Province, China; State Key Laboratory of Microbial Technology, Shandong University, Qingdao 266237, Shandong Province, China; State Key Laboratory of Microbial Technology, Shandong University, Qingdao 266237, Shandong Province, China; State Key Laboratory of Microbial Technology, Shandong University, Qingdao 266237, Shandong Province, China; State Key Laboratory of Microbial Technology, Shandong University, Qingdao 266237, Shandong Province, China; MOE Key Laboratory of Evolution and Marine Biodiversity, Frontiers Science Center for Deep Ocean Multispheres and Earth System & College of Marine Life Sciences, Ocean University of China, Qingdao 266203, Shandong Province, China; Laboratory for Marine Biology and Biotechnology, Qingdao Marine Science and Technology Center & Laoshan Laboratory, Qingdao 266237, Shandong Province, China; Marine Biotechnology Research Center, State Key Laboratory of Microbial Technology, Shandong University, Qingdao 266237, Shandong Province, China; MOE Key Laboratory of Evolution and Marine Biodiversity, Frontiers Science Center for Deep Ocean Multispheres and Earth System & College of Marine Life Sciences, Ocean University of China, Qingdao 266203, Shandong Province, China; School of Life Sciences, University of Warwick, Coventry CV4 7AL, West Midlands, United Kingdom; School of Biosciences, University of Birmingham, Edgbaston B15 2TT, Birmingham, United Kingdom; State Key Laboratory of Microbial Technology, Shandong University, Qingdao 266237, Shandong Province, China

**Keywords:** marine algae, cell wall polysaccharides, Flavobacteriaceae, polysaccharide-degrading enzyme, modular enzyme

## Abstract

Brown algal cell walls are complex matrices composed primarily of alginate, cellulose, and fucoidan. Their depolymerization is important in marine carbon cycling. Although numerous algal polysaccharide-degrading enzymes have been characterized, most studies focus on breaking down single, purified polysaccharides, leaving the degradation mechanisms of native cell walls containing mixed polysaccharides poorly understood. Here, we report the integrated modular enzymes involved in brown algal cell wall polysaccharide (BACWP) degradation. Using the marine flavobacterium *Aquimarina* sp. 2-A2 as a model, we isolated a bifunctional enzyme, CelAly, which integrates a glycoside hydrolase family 5 cellulase domain and a polysaccharide lyase family 31 alginate lyase domain within a single polypeptide, enabling the degradation of cellulose and alginate in brown algal cell walls. *In vivo* relevance of CelAly was confirmed by upregulation of its gene during growth on algal biomass. CelAly also contains three distinctive substrate-binding modules (B1, B2, UKD) that support its multimodular functionality; among these, UKD is notable for its dual substrate-binding capability. CelAly’s modular architecture and interdomain flexibility may facilitate coordinated degradation of BACWPs. Bioinformatic analyses and biochemical validation revealed three additional types of such modular enzymes from marine microbes. CelAly and related modular enzymes are strongly associated with marine environments and exhibit conserved modular strategy for substrate recognition and catabolism. Thus, these enzyme architectures represent a previously unrecognized strategy specialized for BACWP decomposition. This study elucidates the unique structural and functional adaptations of the integrated multimodular enzymes and highlights their ecological prevalence among marine bacteria, providing insights into natural biomass decomposition.

## Introduction

Polysaccharides constitute the primary structural scaffold of plant and algal cell walls, serving as both mechanical barriers and carbon reservoirs for microbial communities [1, 2]. These polysaccharides play essential roles in the global carbon cycle and serve as attractive substrates for biotechnological applications [3, 4]. However, their structural complexity poses a significant challenge for efficient utilisation by microbes. To overcome this structural complexity, microorganisms have evolved sophisticated enzymatic systems mainly relying on diverse carbohydrate-active enzymes (CAZymes) [5, 6]. A prominent example is the efficient multimodular cellulase CelA from *Caldicellulosiruptor bescii*, which combines two catalytic domains with endoglucanase and exoglucanase activities, along with multiple carbohydrate-binding modules (CBMs) [7]. Another case is CbXyn10C/Cel48B, which features a glycoside hydrolase family 10 (GH10) domain (a typical xylanase domain) and a glycoside hydrolase family 48 (GH48) domain (a canonical cellulase domain), both exhibiting bifunctional activity against xylan and cellulose [8]. These examples demonstrate that multimodular CAZymes act as powerful tools for complex terrestrial plant cell wall degradation by combining domains with closely related functional activities.

In marine ecosystems, macroalgae represent major contributors to primary production, releasing vast amounts of organic matter through seasonal blooms and detrital export [9, 10]. These inputs trigger successions of heterotrophic bacteria, with members of Bacteroidota (particularly flavobacteria) acting as dominant degraders [11, 12]. Their ecological success relies on CAZyme-rich genomes, extensive polysaccharide utilization loci (PULs) coupled to SusCD-like uptake systems, and the presence of the type IX secretion system (T9SS) [12, 13]. These organisms therefore play a central ecological role in determining not only the rate of macroalgal carbon turnover but also the chemical form and size distribution of released carbohydrates (e.g. oligosaccharides versus larger particulate fragments), with implications for cross-feeding interactions, microbial loop efficiency, and carbon cycling [14, 15]. Model flavobacteria, such as *Zobellia galactanivorans*, exemplify how these mechanisms support the breakdown of fresh macroalgae, releasing metabolites that serve as public goods for the broader microbial community [12]. However, although we understand how these bacteria process individual polysaccharides [16–19], it remains unclear how they process the intricate physical architecture of the intact cell wall.

The bacterial decomposition of brown algae (*Phaeophyceae*) may serve as a model for studying the bacteria–alga interactions, owing to the high biomass and complex cell wall matrix of brown algae [6, 9]. This matrix consists of alginate [a gel-forming heteropolymer of β-D-mannuronic acid (M) and α-L-guluronic acid (G)] embedding cellulose microfibrils (β-1,4-linked glucan) and other polysaccharides such as fucoidan (a sulfated fucose-rich polysaccharide) [20]. This architecture creates a pronounced accessibility barrier, implying that successful decomposition requires more than isolated single-function CAZymes. Though some specialists, like Verrucomicrobia, manage this by encoding hundreds of enzymes for specific polymers like fucoidan [21], it is unclear whether flavobacteria and even other abundant algal-associated bacteria employ distinct enzymatic strategies to overcome the structural heterogeneity of the entire brown algal cell wall matrix.

In this study, we report the identification and ecological significance of the integrated multimodular enzyme CelAly that enables the coordinated breakdown of native brown algal cell wall polysaccharides (BACWPs). CelAly from the marine flavobacterium *Aquimarina* sp. 2-A2 comprises a cellulase domain (Cel5), an alginate lyase domain (Aly31) and three noncatalytic domains with distinct substrate-binding capacities. *In vitro* and *in vivo* experiments demonstrate that CelAly plays an important role in the bacterial degradation of brown algae. Integrated bioinformatic and biochemical analyses reveal that such CAZymes are particularly abundant in marine flavobacteria and represent an adaptive strategy employed by these bacteria to overcome the structural heterogeneity of algal cell walls, thereby shedding light on microbial degradation of native macroalgal biomass.

## Materials and methods

### Materials and strains

*Aquimarina* sp. 433 was isolated from seaweed collected from seawater in Qingdao, China (120^o^29′42″E, 36^o^5′21″N), and *Aquimarina* sp. 2-A2 was isolated from a sediment sample collected from the Southwest Indian Ocean (50^o^37′56″E, 37^o^34′60″S). Both strains were preserved in our lab, and their genome sequences have been deposited to the GenBank database under accession numbers JBSWZK000000000.1 and JBJKFI000000000.1, respectively. *Escherichia coli* strains were purchased from Vazyme (China). Phosphoric acid swollen cellulose (PASC) and heteropolymers of M and G (PMG) were prepared as previously described [22, 23]. All other carbohydrate substrates were purchased at analytical grade. Detailed information on materials is provided in Supplementary Materials and Methods.

### Real-time quantitative PCR analysis

To verify the role of the multimodular enzyme in brown algae decomposition, the strain *Aquimarina* sp. 433, which harbors the *CELALY’* gene, was cultured in 2216E medium at 30°C to an OD_600_ of 0.8. Cells were collected by centrifugation at 4000 × *g* for 10 min at 4°C, washed three times with a 3% sea salt solution, and then inoculated into the medium containing *Laminaria japonica* and 3% sea salt (pH 8.0). Bacterial samples were collected at 12, 24, and 36 h after inoculation. Total RNA was extracted by using the RNeasy Mini Kit (Qiagen, Germany), followed by cDNA synthesis with the *TransScript* All-in-One First-Strand cDNA Synthesis SuperMix (TransGen, China). RT-qPCR assays were subsequently performed on a LightCycler II 480 System (Roche, Switzerland) using SYBR Premix Ex Taq (TaKaRa, Japan), with the 16S rRNA gene used as the internal reference.

### Gene cloning, mutation, and protein expression, and purification

Genes encoding CelAly (GenBank: WP_407141409.1), its truncated mutations, and selected BACWPs-degrading enzymes (Enzyme A–C) were amplified or synthesized and cloned into the pET-22b vector with a His-tag. Site-directed mutagenesis was performed using the QuikChange kit (Agilent, USA), and the recombinant plasmids were transformed into *E. coli* BL21 (DE3) for protein expression. Recombinant proteins were purified via nickel-affinity and gel filtration chromatography (GE Healthcare, Germany), and protein concentrations were measured using the bicinchoninic acid (BCA) protein assay kit (Thermo, USA). The primers for construction of recCelAly and its truncated mutations are listed in Table S1, and detailed protocols are described in Supplementary Materials and Methods.

### Enzyme assays

The glycoside hydrolase (GH) activities of recCelAly and its mutants, as well as other multimodular enzymes, were determined by the dinitrosalicylic acid (DNS) method [24]. The polysaccharide lyase (PL) activities were determined by the ultraviolet absorption method [25]. After confirming that E173A lacked cellulase activity and Y501A lacked alginate lyase activity, the abilities of both mutants to degrade alginate and cellulose-rich fractions of *L. japonica*, respectively, were assessed using the DNS method [24]. Detailed protocols are described in Supplementary Materials and Methods.

### Biochemical characterization

The optimum temperature, pH, and NaCl concentration for recCelAly, Cel5, and Aly31 activities on carboxymethyl cellulose (CMC) or sodium alginate were determined by individually varying each reaction condition. Degradation products of recCelAly and its mutants toward CMC or sodium alginate were analyzed by high-performance liquid chromatography (HPLC) after 24-h reactions at 40°C. Kinetic parameters for depolymerization of PASC and sodium alginate were determined by nonlinear curve fitting based on the Michaelis–Menten equation using Origin 8.5 software.

### Substrate binding analysis

Insoluble cellulose substrates (PASC, Avicel, and bacterial cellulose) were incubated with proteins, and unbound proteins in the supernatant were quantified by the BCA assay to calculate binding ratios. The UKD domain’s interaction with soluble cellohexaose was analyzed by isothermal titration calorimetry (ITC). Protein binding to alginate substrates, including sodium alginate, PMG, polyguluronate (PG), and polymanuronate (PM), was evaluated by gel filtration chromatography or by incubation with alginate gel beads, with bovine serum albumin (BSA) used as a control. Binding ratios were calculated as the fraction of protein bound relative to the total protein. Detailed protocols are described in Supplementary Materials and Methods.

### Crystallization, data collection, structure determination, and refinement

B1 (the TM4 mutant, 10 mg/ml) was crystallized at 18°C in the buffer containing 0.1 M Bis-Tris propane (pH 8.5) and 28% (w/v) polyethylene glycol (PEG) 6000. UKD (the TM6 mutant, 8 mg/ml) was crystallized in the buffer containing 3.25 M sodium formate (pH 6.8). X-ray diffraction data were collected on the BL02U1 Beamline at Shanghai Synchrotron Radiation Facility (SSRF). The initial diffraction datasets were processed by XDS. Initial model building was finished by the Phenix program AutoBuild [26]. Refinement of the B1 and UKD structures was performed by the Phenix program Refine [26] and Coot [27] alternately. The statistics for data collection and the final model quality are presented in Table S2. All the structure figures were processed using the program PyMOL. The structures of B1 and UKD have been deposited in PDB under identifiers 9KVU and 9KVN, respectively.

### Analysis of alginate assimilation genes

The genomes of strains containing CelAly-like modular enzymes were downloaded from the National Center for Biotechnology Information (NCBI) database and then annotated using the rapid annotation using subsystem technology (RAST) server (http://rast.nmpdr.org/). The prevalence of genes involved in alginate assimilation was statistically analyzed, including *aly* (alginate lyase gene), *oal* (oligoalginate lyase gene), *kdgF* (pectin degradation protein gene), *sdr* (short-chain dehydrogenase/reductase gene), *kdgK* (2-dehydro-3-deoxygluconokinase gene), and *eda* (2-dehydro-3-deoxyphosphogluconate aldolase gene).

### Small-angle X-ray scattering measurement

Small-angle X-ray scattering (SAXS) data for CelAly were collected at SSRF BL19U2 and processed using BioXTAS RAW [28] and ATSAS [29] (Table S3). Scattering profiles were obtained after buffer subtraction. Structural parameters were evaluated using Guinier approximation and GNOM [30]; flexibility was assessed by Kratky plots [31]. *Ab initio* envelopes were generated with DAMMIF [32] and DAMMIN [33]. Rigid-body modeling was performed with CORAL [34] using individual domain models (crystal structures for B1 and UKD and AlphaFold2 predictions for others), with theoretical curves compared to experimental data via CRYSOL [35] and models docked into envelopes using SUBCOMB [36]. Detailed protocols are described in Supplementary Materials and Methods.

### Bioinformatic analysis

The residues encoding a putative signal peptide were predicted by the SignalP 5.0 [37]. Domain analysis was performed in the NCBI Conserved Domain Database (CDD) and dbCAN meta server [38, 39]. Sequence alignments were performed by ClustalW, and alignment figures were processed using WebLogo (http://weblogo.threeplusone.com) [40]. A phylogenetic tree was constructed using the MEGA X software [41] with sequences of related CBMs. Specifically, the tree was constructed through the neighbor-joining approach with a bootstrap analysis of 1000 replicates. The three-dimensional structures of the Cel5, Aly31, and B2 domains of CelAly were predicted by AlphaFold2 with default settings [42], and the models with the highest predicted local distance difference test (pLDDT) scores were selected for further analysis.

## Results and discussion

### Identification of a multimodular enzyme from a marine flavobacterium for efficient degradation of native brown algal cell wall polysaccharides

**Figure 1:**
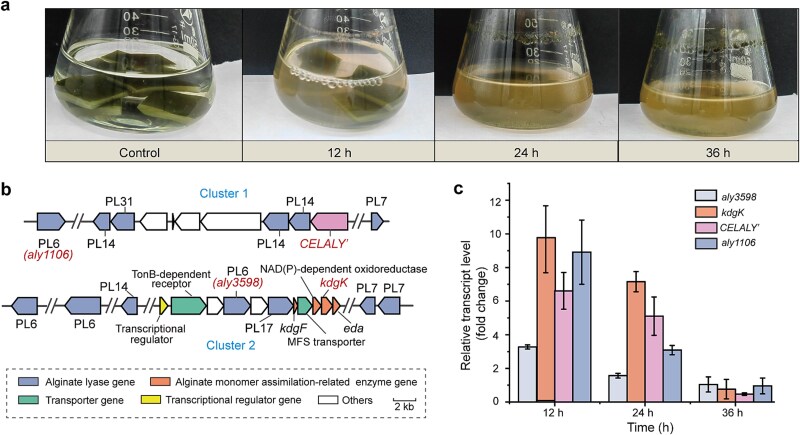
Role of *CELALY’* from *Aquimarina* sp. 433 in brown algae decomposition. (a) Decomposition of brown alga by *Aquimarina* sp. 433. Strain 433 was cultured at 30°C, 180 rpm in the brown alga medium, which contained 4 pieces (1 cm × 1 cm) of *L. japonica* blade and 3% sea salts. Samples were observed at 12, 24, and 36 h post-inoculation. The brown alga medium without strain 433 incubated was taken as the control. (b) Alginate lyase genes and alginate metabolic gene cluster in *Aquimarina* sp. 433. *kdgF*, pectin degradation protein gene, *sdr*, short-chain dehydrogenase/reductase gene. *kdgK*, 2-dehydro-3-deoxygluconokinase gene. *eda*, 2-dehydro-3-deoxyphosphogluconate aldolase gene. (c) RT-qPCR analysis of the transcriptional levels of *CELALY’* and alginate metabolic genes of *Aquimarina* sp. 433 in response to brown alga. The 16S rRNA gene was used as an internal reference. The relative expression levels were normalized to the corresponding gene expression in 2216E medium at the same time points. The graphs show data from triplicate experiments (mean ± SD).

To investigate the enzymatic strategies adopted by marine bacteria for algal biomass decomposition, *Aquimarina* sp. 433, belonging to Flavobacteriaceae, was isolated from seaweed collected from seawater in Qingdao, China (120^o^29′42″E, 36^o^5′21″N). When cultured in artificial seawater supplemented with *L. japonica* (brown alga medium), *Aquimarina* sp. 433 exhibited rapid growth, concomitant with significant degradation of algal biomass, with nearly complete breakdown of the algal substrate observed within 36 h (Fig. 1a), indicating a potent algolytic capability.

Genome analysis revealed that *Aquimarina* sp. 433 encodes 13 putative alginate lyases, likely important for brown algal degradation. Among these, seven are scattered throughout the genome, whereas the remaining six are organized into two distinct gene clusters, designated Cluster 1 and Cluster 2 (Fig. 1b). Cluster 2 resembles a classical alginate utilization locus, containing genes encoding transporters, a regulator, and enzymes involved in monomer assimilation [43, 44] (Fig. 1b). In contrast, Cluster 1 primarily consists of CAZyme-encoding genes (Fig. 1b). Within Cluster 1, we noticed a gene (designated *CELALY’*) that encodes a hexa-modular protein containing a GH family 5 subfamily 2 (GH5_2) domain and a PL family 31 (PL31) domain, as well as 4 other domains. Given that GH5_2 enzymes typically act on cellulose and PL31 enzymes on alginate [45], two important components of brown algal cell walls, this architecture suggested a potential role in concerted cell wall deconstruction. To examine this, we monitor its expression along with that of several other functionally relevant genes during algal decomposition. All tested genes, including *CELALY’*, were upregulated during the early growth phase in brown alga medium, supporting the involvement of *CELALY’* in the degradation process (Fig. 1c).

Due to difficulties in purifying recombinant enzyme encoded by *CELALY’*, we turned to its homolog CelAly from *Aquimarina* sp. 2-A2, which shares 69.0% amino acid sequence identity and retains all conserved domains with CELALY’. *Aquimarina* sp. 2-A2 was isolated from a sediment sample collected from the Southwest Indian Ocean (50^o^37′56″E, 37^o^34′60″S). In contrast with strain 433, strain 2-A2 cannot utilize brown algae for growth. We hypothesize that strain 2-A2 may have lost the metabolic genes during evolution due to the absence of brown algae in its deep-sea habitat. Alternatively, strain 2-A2 may produce CelAly to degrade algal polysaccharides without utilizing the released products, which may instead be assimilated by other bacteria acting as exploiters, thereby facilitating the assembly of polysaccharide-degrading communities [46].

**Figure 2:**
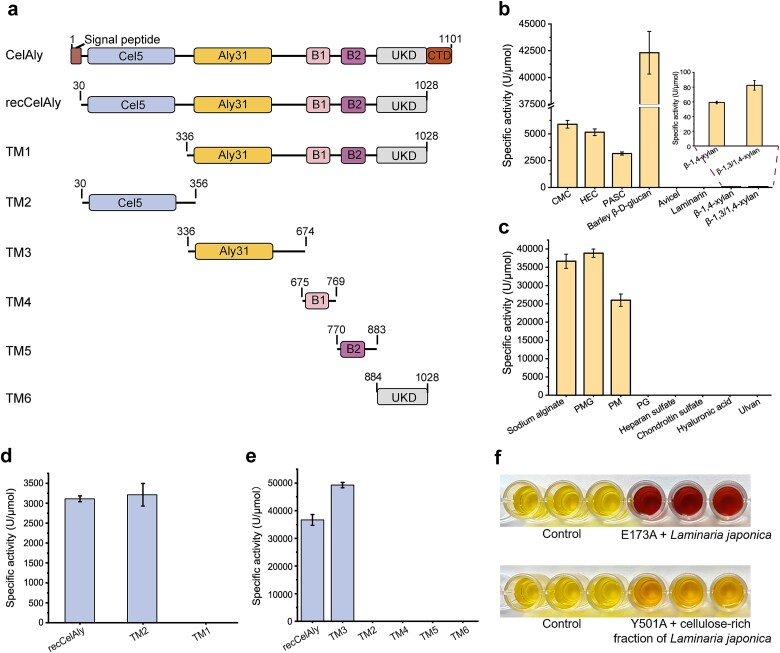
Activities of CelAly toward brown algal cell wall polysaccharides. (a) Schematic diagram of CelAly and its truncated mutants. The signal peptide was predicted by SignalP 5.0 Server. The domain composition was analyzed by the NCBI’s Conserved Domain Database and dbCAN meta server. Cel5, the domain from GH5 family; Aly31, the domain from PL31 family; B1 and B2, two bacterial Ig-like domains of group 7 (Big_7 domains); UKD, an unknown functional domain; CTD, a Por secretion system C-terminal sorting domain. (b) Substrate specificity of recCelAly toward cello- and xylo-configured polysaccharides. CMC, carboxymethyl cellulose. HEC, hydroxyethyl cellulose. PASC, phosphoric acid swollen cellulose. (c) Substrate specificity of recCelAly toward various polysaccharides. PMG, heteropolymers consisting of M and G alternately. PM, polymannuronate. PG, polyguluronate. (d) Cellulase activities of recCelAly and its truncated mutants toward PASC. (e) Alginate lyase activities of recCelAly and its truncated mutants toward sodium alginate. (f) The degradation of *L. japonica* by CelAly mutants. Pictures are representative of three independent experiments. The graphs in (b)–(e) show data from triplicate experiments (mean ± SD).

Consistent with CELALY’, CelAly contains a GH5_2 domain (Cel5) and a PL31 domain (Aly31) (Fig. 2a). In addition, CelAly also contains three conserved domains for which no enzymatic activity has been reported including two bacterial immunoglobulin-like (Big) domains (B1 and B2) and one UKD domain, as well as a Por secretion system C-terminal sorting domain (CTD) domain likely involved in T9SS-dependent secretion (Fig. 2a) [47]. To confirm the function of CelAly, we expressed and purified the recombinant CelAly (recCelAly) lacking the signal peptide and the CTD domain (Fig. S1). A range of polysaccharides known to serve as substrates for GHs and PLs (Table S4) were tested to assess the substrate selectivity of CelAly. recCelAly showed significant GH activity toward barley β-glucan, hydroxyethyl cellulose (HEC), CMC, and PASC but showed much lower activity against xylans (Fig. 2b), indicating that recCelAly functions as a cellulase. For PL-related substrates, recCelAly was exclusively active against alginate substrates, including PM, PMG, and sodium alginate, but not PG (Fig. 2c), indicating that CelAly cleaves the glycosidic bonds between M and G, and M-M linkages but not G-G linkages in alginate. Collectively, these results demonstrate that CelAly is a bifunctional enzyme with both cellulase and alginate lyase activities.

Further domain analysis using a series of truncated mutants (TM1–TM6) (Fig. S1) showed that TM2, containing the Cel5 domain, retained cellulase activity comparable to recCelAly, whereas TM1, which lacked the Cel5 domain, showed no cellulase activity (Fig. 2a and d), confirming that Cel5 is responsible for cellulose degradation. Similarly, TM3 demonstrated alginate lyase activity, whereas the other mutants did not, indicating that Aly31 domain is responsible for alginate degradation (Fig. 2a and e). These results demonstrated that CelAly’s bifunctional activity is driven by its Cel5 and Aly31 domains and that the B1, B2, and UKD domains are not catalytic domains because their removal had minimal impact on substrate specificity (Figs S2a and b), degradation products (Fig. S2c and d), or optimal reaction conditions (Fig. S2e–g).

We further verified CelAly’s activity against native BACWPs *in vitro* using the representative brown alga *L. japonica* as an example. Because *L. japonica* cell walls are rich in alginate but contain low levels of cellulose [48], we performed a sequential degradation assay to distinguish between the two activities. To facilitate this approach, the active-site mutants for both catalytic modules (Cel5: E173A and E259A; Aly31: Y501A and K538A) were constructed based on structural and sequence alignments with their homologs. Activity determination confirmed that E173A and Y501A completely lost the cellulase and alginate lyase activities of recCelAly, respectively (Table S5), thereby enabling selective inactivation of one catalytic module while retaining the activity of the other within the intact multimodular enzyme. In the sequential assay, *L. japonica* was first treated with the mutant E173A to selectively degrade alginate. The generation of reducing sugars was detected by colour development via the DNS method (Fig. 2f). Next, a cellulose-enriched fraction was prepared by removing alginate via hot water extraction, followed by incubation with the Y501A mutant. This treatment also produced significant DNS-reactive products (Fig. 2f). These results demonstrate that CelAly is a BACWPs-degrading enzyme capable of breaking down both alginate and cellulose in brown algal cell walls during algal decomposition.

### Diversity and distribution of CelAly homologs

CelAly shares 61% sequence identity with ClGP from *Cellulophaga lytica* and CelA from *Cellulophaga* sp. RHA 52, both are multimodular enzymes from the ocean (Fig. S3) [49, 50]. ClGP shows cellulase, alginate lyase, and glucuronan lyase activities, whereas CelA exhibits activities toward cellulose, laminarin, and alginate. However, their ability toward native BACWPs has not been reported. Given their sequence similarity to CelAly, it is plausible that they may also function as BACWPs-degrading enzymes. Therefore, the multimodular architecture of CelAly-like enzymes likely represents an adaptive strategy for efficient degradation of BACWPs in natural marine environments.

**Figure 3:**
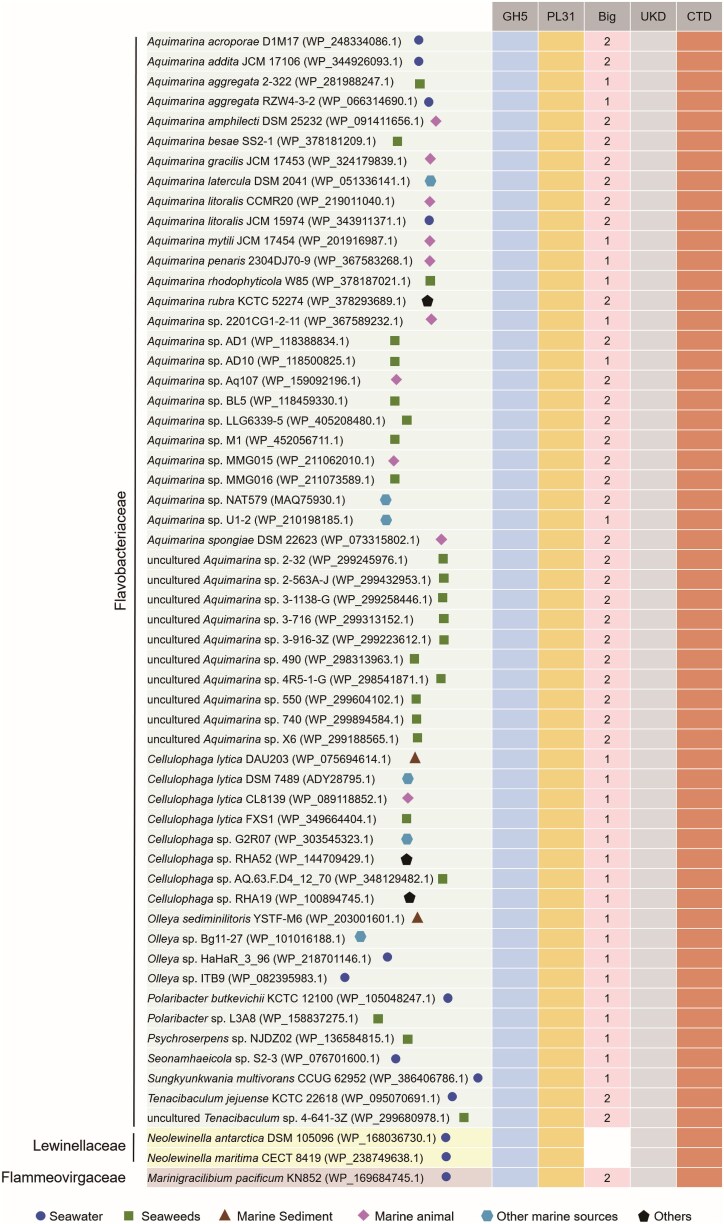
Distribution of CelAly and its homologous proteins in bacteria. The homologs of CelAly were searched against the NCBI nr database using a threshold of ≥75% coverage and ≥40% identity. Domain composition was analyzed using the NCBI Conserved Domain Database (CDD). Filled boxes indicate the presence of a domain, whereas empty boxes indicate its absence. GH5, glycoside hydrolase family 5; PL31, polysaccharide lyase family 31. Big, bacterial Ig-like domain; UKD, domain of unknown function. CTD, a Por secretion system C-terminal sorting domain. The number of Big domains is indicated by the numbers.

To further analyze the ecological significance of such enzymes, we searched the homologs of CelAly against the NCBI nonredundant protein sequences (nr) database (coverage ≥75% and identity ≥40%). A total of 58 homologs were identified, and their conserved domain architectures were further analyzed (Fig. 3). Due to the similarity between domains B1 and B2 that hinders their distinction, both were categorized as Big domains, while the number of Big domains was counted. The result showed that all CelAly homologs contain the catalytic GH5 and PL31 domains, as well as the noncatalytic UKD domain and a T9SS-related CTD domain, with most also carrying one or two Big domains. All the homologs are exclusively within *Bacteroidota*. The majority of these homologs (55/58) are from the *Flavobacteriaceae* family, particularly the genus *Aquimarina*. (36/58), others are from *Marinigracilibium* of *Flammeovirgaceae* (1/58) and *Neolewinella* of *Lewinellaceae* (2/58). These homologs are predominantly marine in origin and enriched in seaweed-associated habitats (Fig. 3), suggesting a key role in BACWPs degradation.

The restricted phylogenetic distribution of CelAly homologs implied that the enzymes may not be a general feature of marine polysaccharide degraders but rather a specialized enzymatic strategy for marine Flavobacteriaceae. Members of *Aquimarina* and related *Flavobacteriaceae* are frequently enriched on macroalgal surfaces and in decaying seaweed biomass [51, 52], environments with high concentrations of structurally intact BACWPs. At such particle-associated niches, efficient access to insoluble, composite substrates is critical for resource acquisition. The conserved fusion of GH5 and PL31 domains may contribute to coordinated depolymerization of cellulose and alginate directly on the algal surface. The universal presence of the CTD domains among CelAly homologs further supports this interpretation because T9SS-mediated secretion and surface anchoring are hallmarks of Bacteroidota specialized in degrading insoluble biopolymers [53]. The restricted taxonomic distribution, conserved multimodular architecture, and functional activity against native BACWPs suggest that CelAly-like enzymes may represent a niche-adapted solution for BACWPs degradation, likely contributing to localized carbon turnover during macroalgal decomposition in marine ecosystems.

### Noncatalytic accessory domains of CelAly show novel polysaccharide-binding mechanisms and enhance CelAly’s affinities for BACWPs

Given that the B1, B2, and UKD domains of CelAly are noncatalytic, we next investigated whether they contribute to the binding of BACWPs. Both domains B1 and B2 specifically interacted with alginate and alginate gel beads but not linear alginate derivatives (PMG, PM, or PG) or the cellulose substrate PASC (Fig. S4a–c), indicating a requirement for a 3D alginate gel network; notably, B1 showed stronger binding (Fig. S4c). Structure analysis revealed that B1 adopts a typical Ig-like β-sandwich fold with a positively charged, flat surface (Fig. S5a and b, Table S2), unlike the weaker-binding B2 (Fig. S5b) and the cleft- or pocket-type alginate-binding CBMs (Fig. S5c) [54–56]. Key residues Lys40^B1^, Arg49^B1^, and Arg75^B1^ (residue numbering refers to positions within the indicated domain) were confirmed to be essential for binding via this binding interface (Fig. S5d–f).

**Figure 4:**
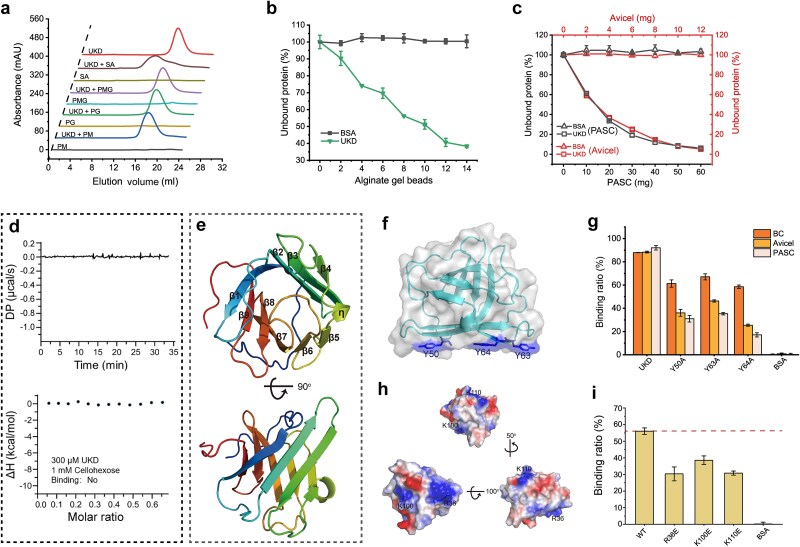
The substrate-binding ability and binding mechanism analyses of the UKD domain. (a) Gel filtration chromatography analysis of the binding ability of the UKD domain toward the alginate-related substrates. SA, sodium alginate. PMG, heteropolymers consisting of M and G alternately. PM, polymannuronate. PG, polyguluronate. (b) Binding ability of the UKD domain toward alginate gel beads. (c) Binding ability of the UKD domain toward phosphoric acid swollen cellulose (PASC) and Avicel. (d) ITC analysis of the binding ability of UKD domain toward cellohexose. Data are representative of three independent experiments. (e) The overall structure of the UKD domain (Cys884-Cys1010) (PDB ID: 9KVN). (f) The key residues involved in cellulose binding in the UKD domain. (g) The cellulose-binding ability of UKD and its mutants toward bacterial cellulose (BC), Avicel, and PASC. (h) The residues involved in alginate binding in the UKD domain. (i) The binding ability of the mutants of the UKD domain toward alginate gel beads. The graphs in (b), (c), (g), and (i) show data from triplicate experiments (mean ± SD), with bovine serum albumin (BSA) serving as the control.

The UKD domain, located immediately downstream of B2 (Fig. 1a), has no previously known function. We analyzed its binding capacity to various polysaccharides and found that it exhibited a distinct substrate binding pattern compared to B1. In addition to selectively binding to sodium alginate and alginate gel beads (Fig. 4a and b), UKD also bound to insoluble cellulose substrates, such as PASC and Avicel (Fig. 4c), but showed no binding to soluble cellohexaose (Fig. 4d). These results suggest that UKD is a unique domain capable of binding both insoluble cellulose and native alginate. Typically, CBMs can only recognize one substrate type (e.g. polysaccharides with identical or structurally similar units) [57]. Thus, UKD represents the first reported instance of a single domain capable of recognizing two chemically distinct polysaccharides. This dual substrate-binding capability, likely an evolutionary adaptation, allows CelAly to efficiently degrade BACWPs by targeting multiple substrates within the complex algal cell walls.

To elucidate the binding mechanisms of the UKD domain to cellulose and alginate, we solved its structure (Table S2), revealing a β-barrel fold comprising nine β-strands and one 3_10_ helix (η) (Fig. 4e), a folding architecture that has never been reported among the structure-solved CBMs [45, 58]. Structural analysis identified a potential planar binding region, including Tyr50^UKD^, Tyr63^UKD,^ and Tyr64^UKD^, which are conserved across UKD homologs (Figs 4f and S6a). Site-directed mutagenesis followed by binding assays with various insoluble cellulose substrates, including bacterial cellulose (BC), Avicel, and PASC, confirmed the importance of these residues (Fig. 4g). Furthermore, the conserved residues across the UKD surface, Arg36^UKD^, Lys100^UKD,^ and Lys110^UKD^, may be involved in alginate binding based on mutagenesis analysis (Figs 4h and i and S6b).

The significance of these binding domains was further illustrated through kinetic assays (Table 1). Compared with recCelAly, the Cel5 and Aly31 variants lacking the three accessory domains showed higher *K*_m_ values on PASC and alginate, respectively, suggesting that the incorporation of these binding modules improves CelAly’s affinity for BACWPs.

**Table 1:** *K*_m_ values of recCelAly, Cel5, and Aly31 domains toward different BACWP substrates.

Substrate	Enzyme	*K*_m_ (mg/ml)
PASC	recCelAly	50.91 ± 4.6
	Cel5	54.68 ± 4.6
Sodium alginate	recCelAly	0.31 ± 0.06
	Aly31	0.71 ± 0.08

### Noncatalytic accessory domains are prevalent in marine flavobacteria

**Figure 5:**
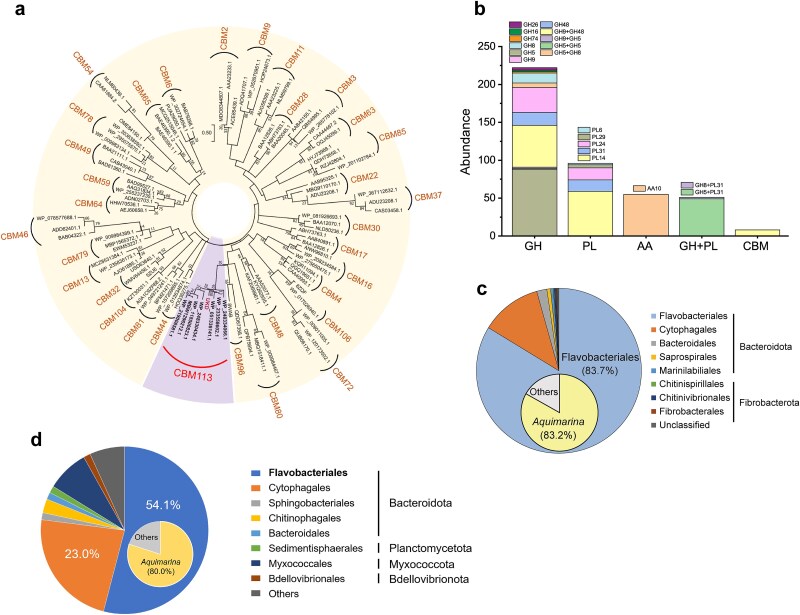
Bioinformatic analyses of the UKD and B1 domains. (a) Phylogenetic analysis of the UKD domain. The phylogenetic tree was constructed using the MEGA X software via the neighbor-joining method. Bootstrap analysis of 1000 replicates was conducted. (b) Distribution of the UKD domain and its homologous proteins in carbohydrate-active enzymes. AA, auxiliary activity. GH, glycoside hydrolase. PL, polysaccharide lyase. GH + PL, enzymes containing GH and PL modules. (c) Taxonomic distribution of UKD homologs in bacteria. (d) Taxonomic distribution of B1 homologs in bacteria. The homologous proteins of UKD and B1 were searched against the NCBI nr database using a threshold of E-value ≤3e-9 and 1e-25, respectively.

Given the unique binding characteristics of UKD compared to previously reported alginate-binding CBMs [54–56, 59–61], we performed comprehensive bioinformatic analysis to determine the relationship of the UKD domain to known CBM families and its distribution across microorganisms. UKD and its homologs formed an independent clade, supporting its classification as the founding member of a new CBM family, CBM113 (Fig. 5a). They share a close phylogenetic relationship with families CBM96 and CBM80, which were reported to bind alginate and to exhibit broad specificity for β-glycans, respectively [55, 62]. A total of 553 UKD homologs were found. More than three-quarters of UKD-containing proteins are predicted to be CAZymes, with GHs (222/432) being the most abundant group. Based on their CAZymes family classification, most of these GHs are glucanase, especially cellulase. The second most common type is PLs (96/432), the majority of which are putative alginate lyases. These are followed by auxiliary activity (AA) enzymes (55/432) and then modular enzymes combining GH and PL domains (51/432). The AA enzymes are exclusively from the AA10 family and are predicted to be lytic cellulose monooxygenases, a class of enzymes crucial for initiating cellulose degradation. The modular enzymes combining GH and PL domains may simultaneously act on cellulose and alginate, akin to CelAly. These findings indicate that the UKD domain frequently co-occurs with enzymes targeting cellulose and alginate (including bifunctional modular enzymes), reinforcing its functional association in the degradation of these polysaccharides (Fig. 5b).

The majority of the UKD homologs belong to the *Bacteroidota* phylum, with a very small number originating from *Fibrobacterota* (Fig. 5c). Within *Bacteroidota*, UKD homologs are predominantly within the *Flavobacteriales* order (83.7%), particularly in the genus *Aquimarina*. The second and third most abundant orders are *Cytophagales* (11.9%) and *Bacteroidales* (2.0%), respectively. We also observed that the distribution of B1 homologs is highly similar to that of UKD. B1 homologs were predominantly in *Bacteroidota* (82.4%), mainly within *Flavobacteriales* (54.1%) and *Cytophagales* (23.0%), with minor occurrences in *Myxococcota, Planctomycetota*, and *Bdellovibrionota* (Fig. 5d).

Although the continuous discovery of novel GHs and PLs from Flavobacteria underscores their quantitative advantage in enzymatic repertoires for algal polysaccharide degradation [63], UKD represents another aspect of adaptation: the evolution of unusual accessory modules that expand polysaccharide recognition. Namely, unlike typical CBMs, which target a single polysaccharide class, UKD exhibits dual-substrate binding. Its frequent co-occurrence with cellulases, alginate lyases, and GH + PL modular enzymes suggest that UKD is a broadly selected functional module for polysaccharide binding. UKD likely enhances enzyme localization on complex algal cell wall matrices, thereby increasing effective substrate concentration and catalytic efficiency in natural environments. In comparison, B1 represents a distinct strategy: rather than inventing a new CBM fold, a ubiquitous Big domain, typically involved in protein stability/adhesion [64, 65], has been repurposed to specifically recognize native alginate via a positively charged planar surface. These findings indicate that marine flavobacteria gain competitiveness not only by expanding CAZyme repertoires but also by diversifying accessory modules, through both the emergence of novel fold and functional rewiring of existing scaffold, supporting efficient degradation of complex BACWPs.

### Diversity and distribution of CelAly-like modular polysaccharide-degrading enzymes

**Figure 6:**
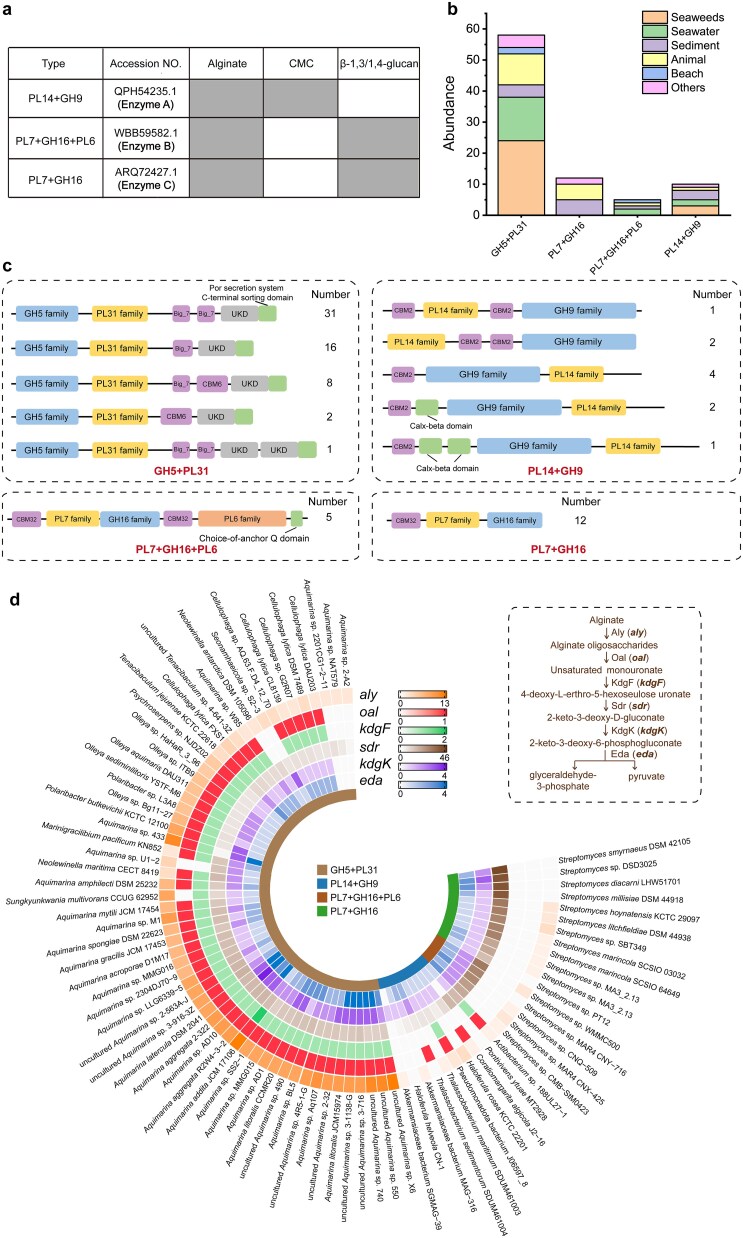
Diversity and distribution of putative BACWP-degrading enzymes. (a) Activities of putative BACWP-degrading enzymes with different domain architectures toward different BACWP substrates. The presence (solid box) or absence (hollow box) of detectable activity is shown. CMC, carboxymethyl cellulose. The putative BACWP-degrading enzymes were searched in the CAZy database and annotated using the dbCAN meta server. (b) The abundance of marine-derived BACWP-degrading enzymes across various marine environments. (c) Schematic diagram of the modular architectures of marine-derived BACWP-degrading enzymes. (d) The prevalence of genes related to alginate assimilation in marine bacterial isolates harboring CelAly-like BACWPs-degrading enzymes. *aly*, alginate lyase gene. *oal*, oligoalginate lyase gene. *kdgF*, pectin degradation protein gene, *sdr*, short-chain dehydrogenase/reductase gene. *kdgK*, 2-dehydro-3-deoxygluconokinase gene. *eda*, 2-dehydro-3-deoxyphosphogluconate aldolase gene. Genes encoding PL15 or PL17 enzymes were classified as *oal*.

Given the potential of modular architectures combining glucan hydrolase and alginate lyase domains to represent a model for enzymes degrading BACWPs, we analysed sequences in the CAZy database from all 13 PL families (PL5, −6, −7, −14, −15, −17, −18, −31, −34, −36, −39, −41, and −44), which are predominantly composed of alginate lyases, and specifically selected those sequences containing additional GH domains. As a result, we systematically identified four distinct enzyme types: GH5 + PL31 (containing both GH5 domain and PL31 domains; including CelAly), PL14 + GH9, PL7 + GH16 + PL6, and PL7 + GH16. To experimentally validate activities beyond that of CelAly (a GH5 + PL31 enzyme), one representative marine-derived sequence from each of the other three types (Enzyme A, B, and C) was selected for enzymatic characterization (Table S6). Enzymes A–C exhibited alginate lyase activity, along with activity against glucan substrates, including β-1,3/1,4-glucan and the cellulose substrate CMC (Fig. 6a). β-1,3/1,4-Glucan has recently been identified as a common component of brown algal cell walls, where it is encapsulated within the alginate matrix [66]. These results confirm their potential as genuine BACWP-degrading enzymes.

Expanding our search using CelAly and Enzymes A–C as query sequences against the NCBI nr database (coverage ≥75%, identity ≥55%) yielded 85 marine-derived homologs, with GH5 + PL31 enzymes showing remarkable predominance (Fig. 6b and Table S6). The limited number is potentially due to the metagenomic assembly biases for multimodular enzymes. Further analysis of their origins revealed that these enzymes are from diverse marine environments, including seaweed, seawater, sediment, animal, and beach (Fig. 6b). Approximately 31.8% of them were isolated from seaweed-associated habitats, suggesting their specialized adaptation to algal polysaccharide degradation in marine ecosystems (Fig. 6b).

Detailed domain architecture analysis revealed distinct, yet convergent evolutionary strategies among the BACWP-degrading enzymes (Fig. 6c). PL7 + GH16 + PL6 enzymes displayed complex CBM32-PL7-GH16-CBM32-PL6 arrangements, whereas PL7 + GH16 enzymes exhibit simpler CBM32-PL7-GH16 architecture, both utilizing the CBM32 domain for alginate recognition [61]. PL14 + GH9 enzymes combine PL14 alginate lyase and GH9 glucanase catalytic domains with one or two CBM2 family domains for cellulose-binding [58]. GH5 + PL31 enzymes showed greater diversity in their auxiliary domains while maintaining a conserved GH5-PL31 catalytic core. The predominant pattern is exemplified by CelAly (31/58), and variants included single Big domains (16/58), Big + CBM6 combinations (8/58), or CBM6 replacements (2/58), with CBM6 known as the cellulose-binding module [58]. The atypical utilization of Big and UKD domains may confer enhanced substrate accessibility to these enzymes during BACWP degradation, thereby explaining their evolutionary advantage and numerical predominance over other types of BACWP-degrading enzymes. These findings reveal that all four types of BACWP-degrading enzyme share a conserved principle for architecture arrangement: catalytically essential domains are consistently paired with specialized binding modules targeting BACWP components. This convergence suggests that diverse microorganisms may evolve similar enzymatic strategies to efficiently degrade BACWPs, highlighting the evolutionary importance of this degradation strategy. Such substrate-driven convergence is not limited to extracellular depolymerases and was also observed in polysaccharide uptake systems, exemplified by the SusCD complexes of Bacteroidota, where SusC (outer-membrane transporter) and SusD (surface glycan-binding protein) homologs cluster by polysaccharide type (e.g. alginate, laminarin) rather than taxonomy [63]. This suggests that structurally complex marine algal polysaccharides impose similar selective pressures across multiple functional layers of utilization systems, from extracellular depolymerization to substrate-specific uptake, thereby facilitating efficient processing of algal polysaccharides in marine environments.

Considering that all the modular enzymes demonstrate alginate-degrading activity, we also analyzed the abundance of genes related to alginate assimilation in the corresponding strains, including alginate lyase genes (*aly*)*,* oligoalginate lyase genes (*oal*), and genes involved in monosaccharide assimilation (Fig. 6d) [43]. In our annotation, alginate lyases from the PL15 and PL17 families were classified as oligoalginate lyases (Oals) because most characterized enzymes in these families exhibit Oal activity [43]. However, Oal activity has also been reported in other PL families. Due to the ambiguity of functional prediction based solely on sequence or family membership, all putative alginate lyase genes from other PL families were conservatively assigned as *aly*. As a result, the number of genes annotated as *oal* is likely an underestimate. Among strains carrying GH5 + PL31-type enzymes, nearly all the alginate assimilation-related genes were detected in the majority of isolates. Among strains harbouring other types of modular enzymes, only a subset of these metabolic genes was identified. However, the reaction catalysed by KdgF can also occur spontaneously [67], and most of these strains still possess multiple alginate lyase genes (Fig. 6d). The absence of certain functional genes may be due to substantial sequence divergence from known references, because these strains (primarily *Actinomycetes*) are phylogenetically distant from the typical sources of reference genes (mostly *Bacteroides*). Collectively, these results suggest that these strains likely have the capacity to assimilate BACWPs, as demonstrated here with alginate, and that CelAly-like modular enzymes in these strains may play an important role in brown algae degradation, akin to the function verified in *Aquimarina* sp. 433.

### BACWP degradation model by CelAly-like modular enzymes

Due to difficulties in determining the complete structure of recCelAly, we employed SAXS to investigate its overall conformation and domain organization (Table S3 and Fig. S7). The *ab initio* beads model revealed an elongated molecular shape with approximate dimensions of 163 × 52 × 60 Å (Fig. S8). Rigid body modelling incorporating the crystal structures of B1 and UKD domains along with predicted models of Cel5, Aly31, and B2 domains supported a side-by-side domain arrangement (Figs S8 and S9a). The Kratky plot displayed an upward trend at high *q* values, indicating the presence of partial disorder and flexible linkers between domains (Fig. S9b) [68].

Based on the structural and biochemical results, we propose the putative mechanism for BACWP degradation by CelAly. The process initiates with binding of the B1 and UKD domains to the alginate matrix through distinct binding mechanisms, enhancing substrate affinity and positioning Aly31 for efficient alginate cleavage. As alginate degradation progresses, exposed cellulose microfibrils become accessible for UKD binding, which subsequently positions Cel5 for cellulose hydrolysis. The proximity of B1 and Aly31 mirrors the canonical CBM-catalytic domain arrangement observed in other highly efficient CAZymes [69]. In contrast, the spatial separation of the UKD and two catalytic domains (Cel5 and Aly31) likely allow dynamic interactions. When UKD binds alginate, it may lead to conformational changes that optimize Aly31’s access to alginate while prepositioning Cel5 near emerging cellulose microfibrils. The flexible interdomain architecture might allow Cel5 to hydrolyze cellulose while Aly31 continues processing adjacent alginate regions. This coordinated action is mediated by UKD, which functions as a “hook” that captures substrates and orchestrates catalytic domain positioning.

**Figure 7:**
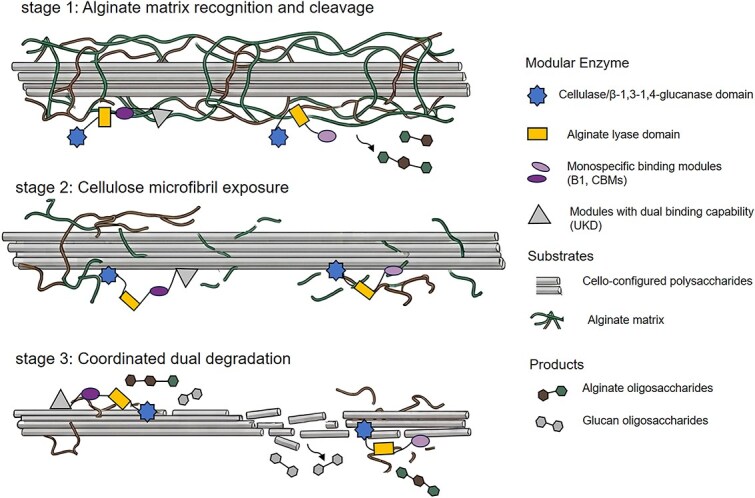
The mechanistic paradigm for modular enzymes on BACWPs. The modular enzyme degrades BACWPs through a three-stage process. (1) Alginate recognition and cleavage: Alginate-specific binding modules and modules with dual binding capability recognize and anchor onto the alginate matrix, enabling the alginate lyase domain to initiate depolymerization. (2) Cellulose microfibril exposure: Degradation of the alginate matrix exposes embedded cello-configured polysaccharides., which allows adaptive repositioning of binding and catalytic modules to the changing substrate landscapes. (3) Coordinated dual degradation: Facilitated by the substrate binding from both alginate/cello-specific modules and modules with dual substrate-binding capability, the alginate lyase and cellulase domains degrade both exposed substrates to generate alginate and glucan oligosaccharides. Flexible linkers ensure optimal domain organization throughout the dynamic degradation process.

Extending from CelAly to the various BACWP-degrading enzymes analysed in the earlier section, we propose a generalized mechanistic paradigm for their action on BACWPs (Fig. 7). This mechanism involves three key elements: (i) specialized binding modules for cello-configured polysaccharides and/or alginate substrate recognition, including both monospecific modules (e.g. B1 and CBMs) and dual-binding specific modules (e.g. UKD); (ii) polysaccharide degradation through coordinated action of at least two catalytic domains targeting on different types of substrates; and (iii) dynamic structural coordination mediated by flexible linkers. In this system, the enzymes may first target the protective alginate matrix, thereby exposing the underlying cello-configured polysaccharides, after which both alginate and cello-configured substrates can be degraded in a coordinated manner. The flexible interdomain organization might allow adaptive repositioning of catalytic modules during this degradation process primarily mediated by the binding modules.

Though some multimodular CAZymes have been reported for the degradation of terrestrial plant polysaccharides, most of the enzymes combine domains that target a single polysaccharide type (e.g. cellulose or hemicellulose) [7, 70]. Distinct from these enzymes, CelAly-like enzymes display a unique catalytic domain architecture and distinct auxiliary modules. Through the coordinated action of these functionally diverse catalytic domains and auxiliary modules, the degradation of native BACWPs is achieved, underscoring its more sophisticated enzymatic digestion strategy specialized for algal metabolism. This mechanistic framework provides insights into the bacterial evolution of multifunctional enzymes for algal biomass degradation.

## Conclusion

Brown algal biomass represents one of the largest pools of marine primary production, yet the enzymatic strategies enabling its efficient microbial decomposition remain incompletely understood, particularly given the composite nature of BACWPs. Here, we identify and characterize a previously underappreciated type of multimodular enzyme specialized in the coordinated degradation of BACWPs, exemplified by CelAly-like enzymes from marine Flavobacteriaceae. CelAly integrates a cellulase and an alginate lyase within a single protein, each independently contributing to the depolymerization of cellulose and alginate. Degradation assays using *L. japonica* confirm its effectiveness against native brown algal tissues. Noncatalytic accessory domains of CelAly are important for efficient degradation of native BACWPs. The B1 domain selectively recognizes alginate gels through a positively charged planar interface, and the UKD domain possessing a novel fold that binds both insoluble cellulose and native alginate. These modules increase substrate affinity and suggest a mechanistic basis for efficient enzyme localization and coordinated catalysis on complex cell wall assemblies. Genomic surveys reveal that CelAly homologs and associated accessory domains are largely restricted to marine Flavobacteriaceae, and that such GH + PL modular architectures are enriched in strains from seaweed-associated habitats.

Together, the findings provide insights into how marine bacteria access and degrade structurally complex macroalgal cell walls. Rather than relying solely on collections of single-function CAZymes, CelAly-like enzymes represent an integrated strategy that targets multiple key BACWP components within the same physical matrix. The presence of specialized binding modules further suggests that efficient degradation depends not only on catalytic capacity but also on the ability of extracellular enzymes to attach to heterogeneous polysaccharide assemblies. Such features likely affect the efficiency of algal tissue breakdown and the forms of released carbohydrates available to other microbes. The strong phylogenetic restriction of these enzymes to marine Bacteroidota indicates that these lineages are adapted for macroalgal polysaccharide degradation. By generating accessible substrates, such degraders may facilitate downstream trophic interactions and carbon flow through microbial consortia. Overall, this study links multimodular enzyme architecture to ecological strategy, providing mechanistic insight into how specific bacterial groups contribute to macroalgal biomass processing. By converting particulate matter into soluble carbohydrates, these enzymatic systems may contribute to carbon cycling in marine ecosystems.

## Supplementary Material

supplementary_materials_for_revision-2_wrag112

## Data Availability

The genomic data of *Aquimarina* sp. 433 and *Aquimarina* sp. 2-A2 are available in NCBI under the accession numbers JBSWZK000000000.1 and JBJKFI000000000.1, respectively. The structures of B1 and UKD have been deposited in PDB under identifiers 9KVU and 9KVN, respectively.
