# Supplementary material for: Multimodular flavobacterial enzymes specialized in coordinated decomposition of cellulose and alginate in brown algal cell walls
Source: ISME J. 2026 May 8;20(1):wrag112. doi: 10.1093/ismejo/wrag112 (PMC13235733; doi:10.1093/ismejo/wrag112)
Supplement: supplementary_materials_for_revision-2_wrag112 [file supplementary_materials_for_revision-2_wrag112.docx]

**Supplementary Materials for**

**Multimodular flavobacterial enzymes specialised in coordinated decomposition of cellulose and alginate in brown algal cell walls**

**This PDF includes** supplementary materials and methods, Figures S1 to S9, and Tables S1 to S6.

**Supplementary Materials and Methods**

**Materials.** Carboxymethyl cellulose (CMC), hydroxyethyl cellulose (HEC), Avicel, laminarin, and cellobiose were purchased from Sigma (USA). Barley β-glucan (β-1,3/1,4-glucan), beechwood xylan (β-1,4-xylan), and cellotriose were purchased from Megazyme (Ireland). Xylan from *Palmaria palmata* (β-1,3/1,4-xylan) and ulvan were purchased from Elicityl (France). Bacterial cellulose (BC) dispersion was purchased from Guilin Qihong Technology Co., Ltd (China). Sodium alginate and glucose were purchased from Sinopharm Chemical Reagent Co., Ltd (China). Polymannuronate (PM), polyguluronate (PG) and mannuronate oligomers were purchased from Shanghai Zzbio Co., Ltd. (China). Hyaluronic acid was purchased from Shanghai Macklin Biochemical Co., Ltd (China). Chondroitin sulfate was purchased from Shanghai yuanye Bio-Technology Co., Ltd (China). Heparan sulfate was purchased from Nanjing Shenglide Biotechnology Co., Ltd (China).

**Gene cloning, mutation, and protein expression and purification.** The genes encoding CelAly (Genbank: WP_407141409.1) and its truncated mutations were amplified via PCR from the genomic DNA of *Aquimarina* sp. 2-A2 with the designed primer pairs (Table S1). The genes of the selected putative BACWPs-degrading enzymes (Enzyme A-C) were synthesized by the TsingkeBiotechnology Co., Ltd (China). The obtained genes were ligated into the pET-22b expression vector between the restriction sites NdeI and XhoI along with a C-terminal His-tag. Site-directed mutagenesis was performed using the QuikChange kit (Agilent, USA). The resulting recombinant vectors were transferred into *Escherichia coli* BL21 (DE3) for heterologous protein expression. The recombinant *E. coli* BL21 (DE3) cells were cultured in Lysogeny broth (LB) medium supplemented with 100 μg/ml ampicillin at 37^o^C. When the OD_600_ value of the culture reached 0.6 - 0.8, protein expression was induced by the addition of 0.3 mM isopropyl β-D-thiogalactopyranoside (IPTG), and the culture was further incubated at 18^o^C for an additional 18 h. After cultivation, bacterial cells were harvested by centrifugation and disrupted by high-pressure cell disruptor in the buffer containing 50 mM Tris-HCl (pH 8.0) and 100 mM NaCl. The recombinant proteins were then purified by nickel-nitrilotriacetic acid resin (GE healthcare, Germany) and gel filtration chromatography (GE healthcare, USA). The concentrations of the recombinant proteins were determined by the bicinchoninic acid (BCA) protein assay kit (Thermo, USA).

**Enzyme assays.** The glycoside hydrolase (GH) activities of recCelAly, the mutants of recCelAly and other multimodular enzymes were determined by the dinitrosalicylic acid (DNS) method [1]. In detail, the enzyme activities of recCelAly and its mutants on glucans (cello-configured glucans: CMC, HEC, Avicel, and phosphoric acid swollen cellulose (PASC); other types of glucans: laminarin and barley β-glucan) and xylans (beechwood xylan and xylan from *Palmaria palmata*) were assessed in a 200-μl reaction system containing 10 mg/ml of each substrate (75 mg/ml for PASC, wet weight), enzyme at an appropriate concentration (1 nM - 5 μM for different substrates), and 50 mM Tris-HCl (pH 7.0) at 50^o^C for 15 min. The GH activities of multimodular enzymes on CMC, laminarin, barley β-glucan were determined in a 200-μl reaction system containing 10 mg/ml of each substrate, enzyme at an appropriate concentration and 50 mM Tris-HCl (pH 7.0) at 30^o^C. After 15 min of incubation, the reactions were terminated with 100 μl DNS reagent, and the mixtures were boiled for colouring. Then, the absorbance at 540 nm was determined. One unit (U) of enzyme activity was defined as the amount of enzyme required to produce 1 μmol of reducing sugars (glucose equivalent) per min.

The polysaccharide lyase (PL) activities of recCelAly and its mutants were determined by the ultraviolet absorption method [2]. In detail, the enzyme activities of recCelAly on various polysaccharides (sodium alginate, heteropolymers of M and G (PMG), PM, PG, heparan sulfate, chondroitin sulfate, hyaluronic acid, or ulvan) were determined in a 200-μl reaction system containing 2 mg/ml substrate, enzyme at an appropriate concentration, 50 mM Tris-HCl (pH 8.0) and 0.5 M NaCl at 60^o^C. The alginate activities of other multimodular enzymes on alginate were measured in a 200-μl reaction system containing 2 mg/ml of alginate, enzyme at an appropriate concentration, and 50 mM Tris-HCl (pH 8.0) at 30^o^C. After 15 min of incubation, the reactions were terminated by boiling the mixture for 10 min. Then, the increase in the absorbance at 235 nm of the mixture was monitored using a Jasco V-550 spectrophotometer (Jasco, Japan). One unit (U) of enzyme activity was defined as the amount of enzyme required to induce an increase of 0.1 at 235 nm per min.

The degradation of alginate in *Laminaria japonica* by E173A and cellulose-rich fraction in *L. japonica* by Y501A were determined using the DNS method [18], following confirmation that E173A lacked cellulase activity and Y501A lacked alginate lyase activity. For E173A-mediated degradation, a 300-μl degradation reaction containing 10 mg/ml *L. japonica* powder, 15 μM mutant E173A and 50 mM Tris-HCl (pH 8.0) was carried out at 40^o^C for 20 min. For Y501A-mediated degradation of the cellulose-rich fraction, a 300-μl reaction system containing 20 mg/ml cellulose-rich fraction of *L. japonica* powder, 15 μM mutant Y501A and 50 mM Tris-HCl (pH 7.0) was incubated at 40°C for 3 h. After incubation, the reaction mixture was centrifuged at 12,000 × *g* for 15 min. Then, 200 μl supernatant was mixed with 100 μl DNS, and the mixtures were boiled for colouring. The control group was performed with the pre-heated inactivated enzyme under the same conditions. By comparing the colour differences between the experimental and control groups, the ability of mutants E173A and Y501A to degrade alginate and cellulose in *L. japonica*, respectively, was determined.

**Biochemical characterization.** The optimum temperature for recCelAly activity toward CMC was determined at a range of 20 to 70^o^C in 50 mM Tris-HCl (pH 7.0), and the optimum temperature toward sodium alginate was determined at a range of 30 to 80^o^C in 50 mM Tris-HCl (pH 8.0) and 0.5 M NaCl. The optimum pH for CelAly activity toward CMC or sodium alginate was determined in the Britton-Robinson (B-R) buffer ranging from pH 5.0 to 10.0. B-R buffer was prepared according to the previously reported method [3]. The effect of NaCl concentration on recCelAly activity toward CMC or sodium alginate was investigated across a range of NaCl concentrations, varying from 0 to 1.75 M for CMC and 0 to 2 M for sodium alginate. The optimum reaction conditions of Cel5 (the TM2 mutant) and Aly31 (the TM3 mutant) were determined by the same methods as for recCelAly.

To determine the degradation products of recCelAly or Cel5 toward CMC, a reaction system containing 5 μM enzyme, 10 mg/ml CMC and 50 mM Tris-HCl (pH 7.0) was carried out at 40^o^C for 24 h. The degradation products were then analysed by high performance liquid chromatography (HPLC) on a Superdex Peptide 10/300 GL column (GE Healthcare, USA) using deionized water as the running buffer. The degradation products were eluted with a flow rate of 0.3 ml/min and monitored by a refractive index detector. Online monitoring and data analysis were conducted using LabSolutions software [4]. The degradation products of recCelAly or the Aly31 domain toward sodium alginate were also analysed by HPLC. The reaction was carried out at 40^o^C for 24 h in the mixture containing 5 μM enzyme, 2 mg/ml sodium alginate, 50 mM Tris-HCl (pH 8.0) and 0.5 M NaCl. When analyzing the degradation products by HPLC, NH_4_HCO_3_ at a concentration of 0.2 M was used as the running buffer, and elution was monitored at 210 nm by a UV detector.

The kinetic parameters of recCelAly or the Cel5 domain for depolymerization of PASC, as well as recCelAly or the Aly31 domain for depolymerization of sodium alginate, were determined by nonlinear curve fitting based on the Michaelis-Menten equation using Origin 8.5 software [5, 6]. The initial reaction rates were assayed across substrate concentration of 12.5 to 200 mg/ml for PASC and 0.1 to 3 mg/ml for sodium alginate.

**Substrate binding analysis.** To assess the binding capacity of the proteins to insoluble cellulose substrates, different proteins at 45 μM were incubated with the substrates (PASC, Avicel, or BC dispersion) in a 200 μL mixture containing 0.2 M phosphate buffer (Na_2_HPO_4_-NaH_2_PO_4_, pH 7.0). The bovine serum albumin (BSA) was used as a control under identical conditions. After incubation at 4^o^C for 5 h with occasional stirring, the mixtures were centrifuged at 10,000 × *g* for 10 min at 4^o^C. The protein content in the supernatants, representing unbound proteins, was quantified using the BCA method. The binding ratio is calculated by the following equation:

Binding ratio (%) = (M_a_ − M_b_) / M_a_ × 100 (1)

where M_a_ is the total protein content added in the binding system, and M_b_ is the unbound protein.

The binding capacity of the UKD domain to soluble cellohexaose was analyzed by isothermal titration calorimetry (ITC) using MicroCal PEAQ-ITC (Malvern, UK) at 25^o^C. The UKD domain and cellohexaose were prepared at concentrations of 0.3 mM and 1 mM, respectively, in 10 mM Tris-HCl (pH 7.0) containing 0.1 M NaCl. Cellohexaose was titrated into the protein cell 13 times with a stirring speed of 750 rpm. The data were analysed with Microcal PEAQ-ITC analysis software.

The binding capacity of the proteins to alginate substrates, including sodium alginate, PMG, PG, and PM, was analysed by gel filtration chromatography. Each protein (150 μM) was mixed with 4 mg/ml of the respective alginate substrates in 0.2 M phosphate buffer (pH 7.0) and incubated on ice for 5 h. The substrate-protein mixtures were then subjected to the Superdex G75 column to determine the elution positions of the proteins. The binding capacity of the B1, B2, and UKD domains to alginate was also analysed by incubating these proteins (45 μM) with varying numbers of alginate gel beads in 0.2 ml of 0.2 M phosphate buffer (pH 7.0), with BSA serving as a control. The alginate gel beads were prepared as previously described [7]. After incubation at 4^o^C for 5 h with occasional stirring, the amounts of unbound proteins in the supernatants were measured by the BCA method. The binding ratio is calculated according to the equation (1).

The binding capacity of the B1 domain to other gel-forming polysaccharides, including agar, carrageenan, and pectin, was analysed by the same method as for alginate substrates using gel filtration chromatography.

**Small-angle X-ray scattering (SAXS) measurement.** SAXS data for CelAly were collected on the BL19U2 beamline of SSRF with a PILATUS 1 M detector (DECTRIS, Switzerland) (Table S3). CelAly solutions (0.5 to 10 mg/mL in 10 mM Tris-HCl, pH 8.0, 100 mM NaCl) were robotically loaded into a 1.5 mm quartz capillary, with 20 independent 1 s exposures recorded at 10^o^C. All SAXS data were processed by BioXTAS RAW [8] and ATSAS software package [9], and protein scattering profiles were obtained after subtracting the buffer profile. The forward scattering (*I*(0)) and the radius of gyration (*R*_g_) were evaluated via the Guinier approximation. The maximum dimension (*D*_max_) and the interatomic distance distribution function (*P*(r)) were computed with the program GNOM [10]. The Kratky plot was used to assess CelAly flexibility [11]. *Ab initio* molecular envelopes were generated and refined by the program DAMMIF [12] and DAMMIN [13]. Rigid body model construction to the experimental scattering data was performed with CORAL [14] using CelAly’s individual domain models. Except for the crystal structures of the B1 and UKD domains, other domain models were predicted by Alphafold2 [15]. The theoretical scattering curve from the atomic model was calculated and compared with the experimental curve by CRYSOL [16]. Atomic model was docked into *ab initio* envelope with the program SUBCOMB [17].

**References**

1. Miller GL. Use of dinitrosalicylic acid reagent for determination of reducing sugar. *Anal Chem* 1959;**31**:426-8.

2. Xu F, Chen XL, Sun XH. *et al*. Structural and molecular basis for the substrate positioning mechanism of a new PL7 subfamily alginate lyase from the arctic. *J Biol Chem* 2020;**295**:16380-92.

3. Wang Y, Liu BX, Cheng JH. *et al*. Characterization of a new M4 metalloprotease with collagen-swelling ability from marine *Vibrio pomeroyi* strain 12613. *Front Microbiol* 2020;**11**:1868.

4. Wang Y, Jiang W, Zhang Y. *et al*. Structural insight into chitin degradation and thermostability of a novel endochitinase from the glycoside hydrolase family 18. *Front Microbiol* 2019;**10**:2457.

5. Sun X, Zhang X, Zhang X. *et al*. Direct preparation of alginate oligosaccharides from brown algae by an algae-decomposing alginate lyase AlyP18 from the marine bacterium *Pseudoalteromonas agarivorans* A3. *Mar Drugs* 2024;**22**:483.

6. Zhao F, Cao HY, Zhao LS. *et al*. A novel subfamily of endo-beta-1,4-glucanases in glycoside hydrolase family 10. *Appl Environ Microbiol* 2019;**85**:e01029-19.

7. Tang L, Guo E, Zhang L. *et al*. The function of CBM32 in alginate lyase VxAly7B on the activity on both soluble sodium alginate and alginate gel. *Front Microbiol* 2021;**12**:798819.

8. Hopkins JB, Gillilan RE, Skou S. *BioXTAS RAW*: improvements to a free open-source program for small-angle X-ray scattering data reduction and analysis. *J Appl Crystallogr* 2017;**50**:1545-53.

9. Manalastas-Cantos K, Konarev PV, Hajizadeh NR. *et al*. *ATSAS 3.0*: expanded functionality and new tools for small-angle scattering data analysis. *J Appl Crystallogr* 2021;**54**:343-55.

10. Svergun DI. Determination of the regularization parameter in indirect-transform methods using perceptual criteria. *J Appl Crystallogr* 1992;**25**:495-503.

11. Kikhney AG, Svergun DI. A practical guide to small angle X-ray scattering (SAXS) of flexible and intrinsically disordered proteins. *FEBS Lett* 2015;**589**:2570-77.

12. Franke D, Svergun DI. *DAMMIF*, a program for rapid *ab-initio* shape determination in small-angle scattering. *J Appl Crystallogr* 2009;**42**:342-6.

13. Svergun DI. Restoring low resolution structure of biological macromolecules from solution scattering using simulated annealing. *Biophys J* 1999;**76**:2879-86.

14. Petoukhov MV, Franke D, Shkumatov AV. *et al*. New developments in the ATSAS program package for small-angle scattering data analysis. *J Appl Crystallogr* 2012;**45**:342-50.

15. Jumper J, Evans R, Pritzel A. *et al*. Highly accurate protein structure prediction with AlphaFold. *Nature* 2021;**596**:583-9.

16. Svergun D, Barberato C, Koch MHJ. *CRYSOL*- a program to evaluate X-ray solution scattering of biological macromolecules from atomic coordinates. *J Appl Crystallogr* 1995;**28**:768-73.

17. Kozin M, Svergun D. Automated matching of high- and low- resolution structural models. *J Appl Crystallogr* 2001;**34**:33-41.


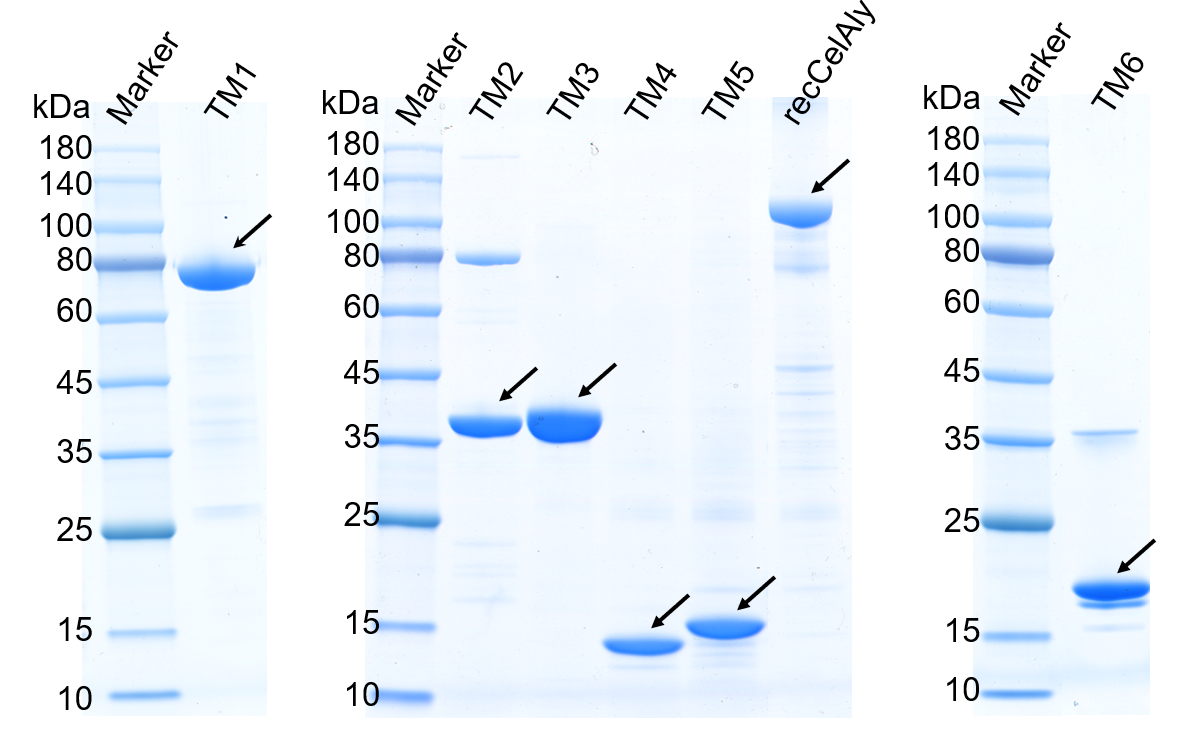


**Figure S1. SDS-PAGE analysis of the purified recCelAly and its truncated mutants.**

**
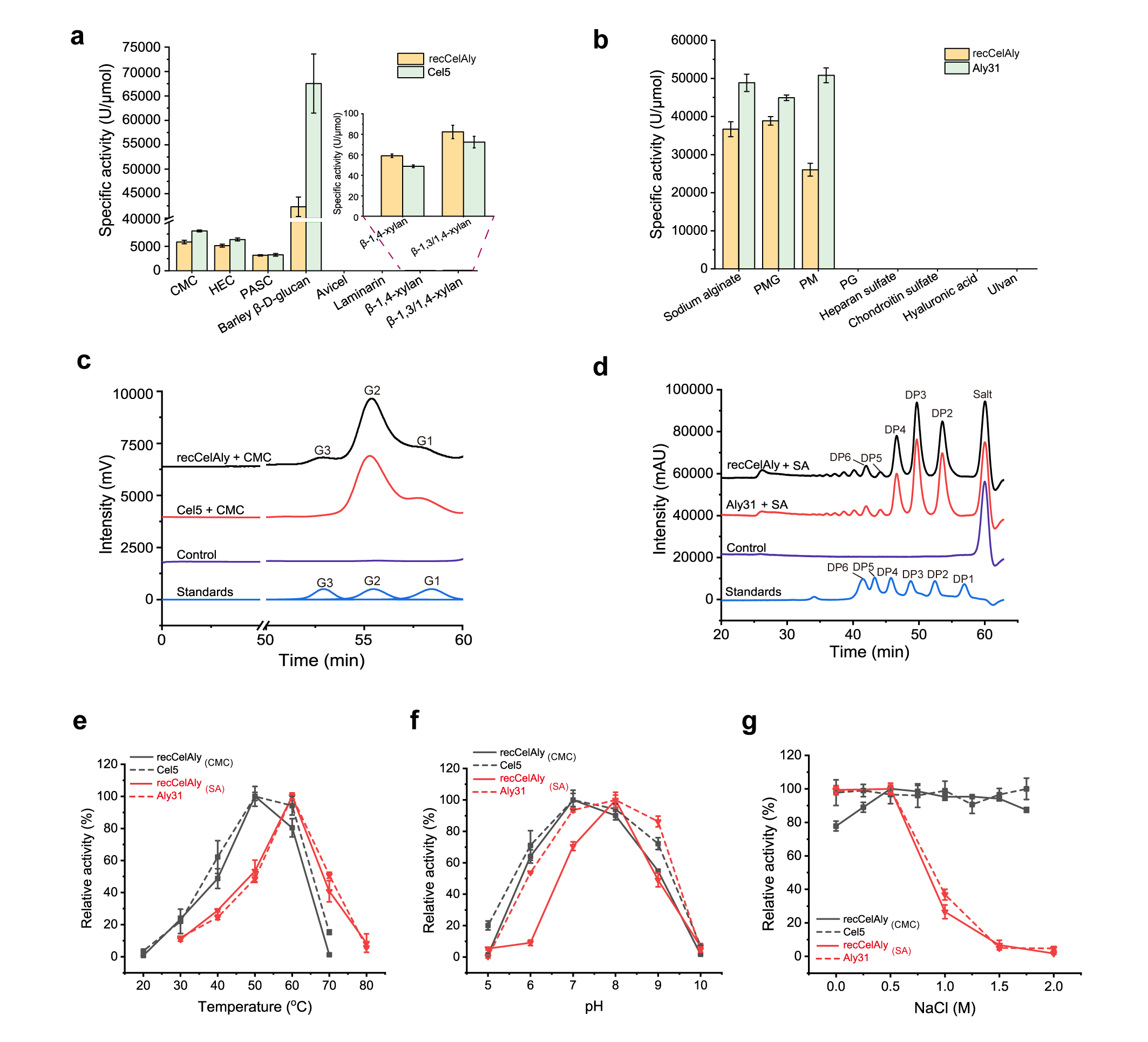
Figure S2. Characterization of recCelAly and its catalytic domains Cel5 and Aly31.** (a) Substrate specificity of recCelAly and Cel5 toward glucans and xylan substrates. CMC, carboxymethyl cellulose. HEC, hydroxyethyl cellulose. PASC, phosphoric acid swollen cellulose. (b) Substrate specificity of recCelAly and Aly31 toward various polysaccharides. PMG, heteropolymers consisting of M and G alternately. PM, polymannuronate. PG, polyguluronate. (c) The degradation products of recCelAly and the Cel5 domain toward carboxymethyl cellulose analyzed by HPLC. CMC, carboxymethyl cellulose. G1, glucose; G2, cellobiose; G3, cellotriose. (d) The degradation products of recCelAly and the Aly31 domain toward sodium alginate analyzed by HPLC. SA, sodium alginate. DP, degree of polymerization. Mannuronate oligomers from DP1 to DP6 were taken as the standards. The control groups in (c) and (d) were treated with preheated inactivated enzymes, and data are representative of three independent experiments. (e) - (g) Effects of temperature (e), pH (f) and NaCl concentration (g) on enzyme activity of recCelAly, Cel5, and Aly31 domains. The graphs in (e) - (g) show data from triplicate experiments (mean ± SD).


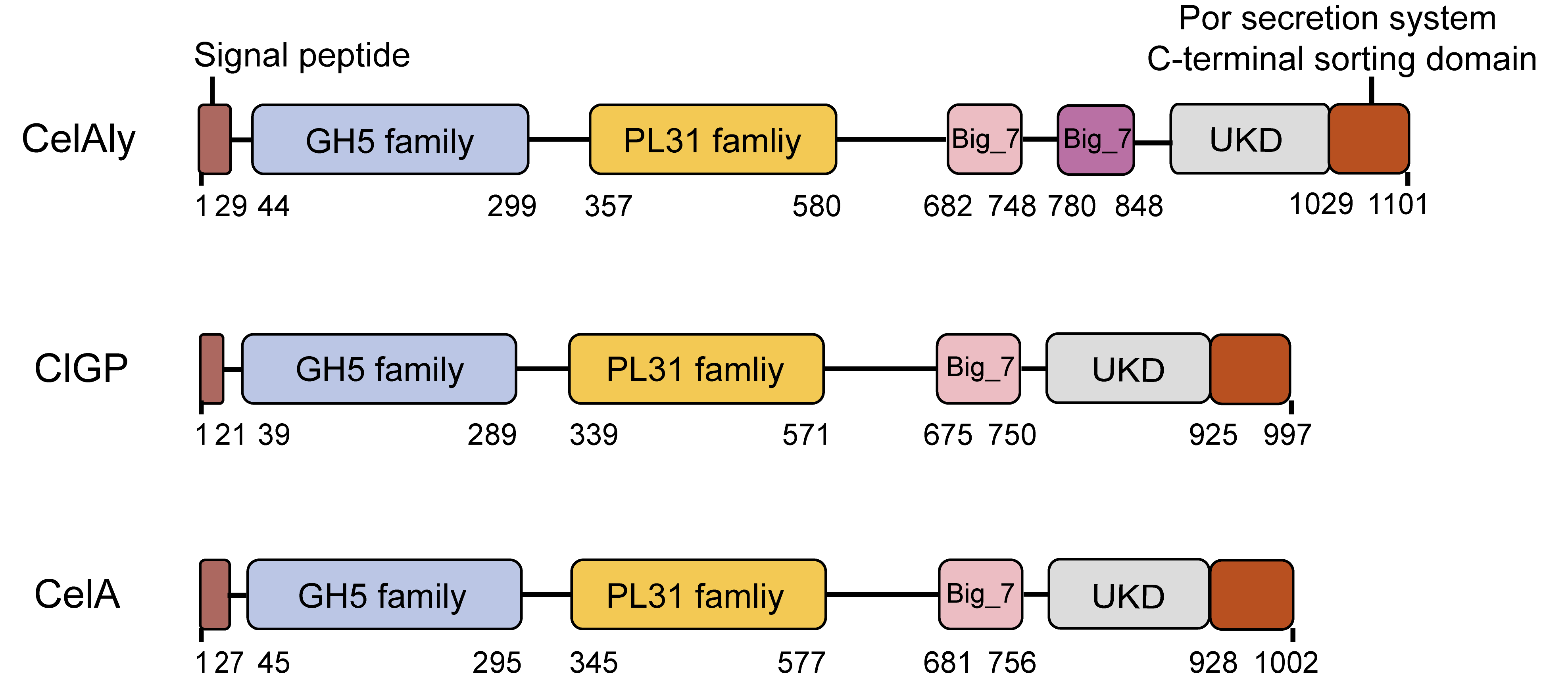


**Figure S3. Domain structure comparison between CelAly and the two reported multimodular enzymes.**

**
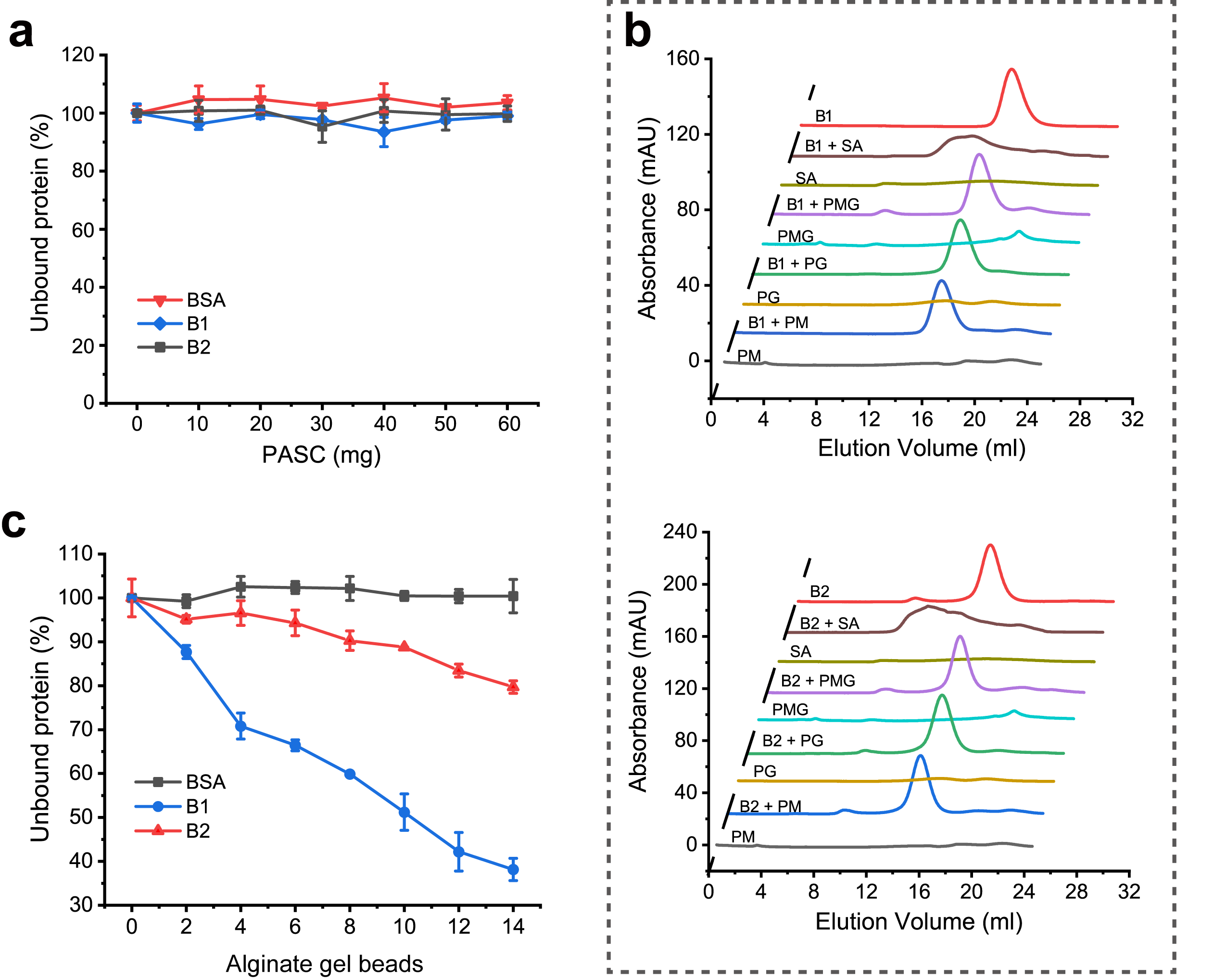
**

**Figure S4. The substrate binding ability of the B1 and B2 domains.** (a) Binding ability of the B1 and B2 domains toward PASC. (b) Gel filtration chromatography analysis of the binding ability of the B1 and B2 domains toward the alginate-related substrates, including sodium alginate (SA), PMG, PG, and PM. (c) Binding ability of the B1 and B2 domains toward alginate gel beads. The graphs in (a) and (c) show data from triplicate experiments (mean ± SD), and data in (b) are representative of three independent experiments.

**
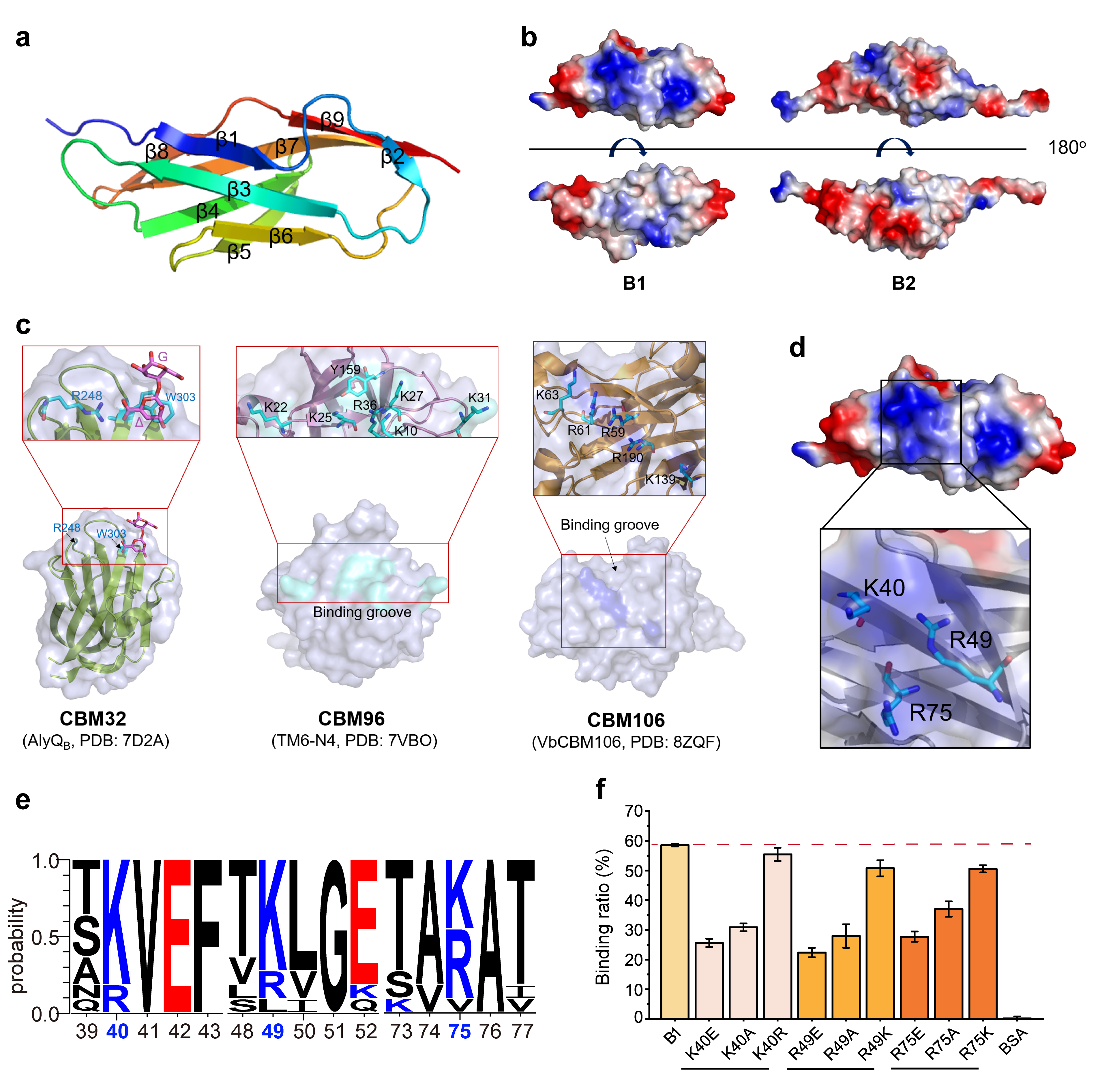
**

**Figure S5. Binding mechanism of the B1 domain.** (a) Overall structure of the B1 domain (Gly675-Gly769) (PDB ID: 9KVU). (b) Electrostatic surface views of the B1 and B2 domains. The structure of the B2 domain (Asn775-Thr877) was predicted by AlphaFold2. (c) The binding sites of the reported alginate-binding domains AlyQ_B_, TM6-N4, and VbCBM106. Residues involved in alginate binding are shown as cyan sticks. G, guluronate. Δ, the unsaturated monosaccharide (4-deoxy-L-erythro-hex-4-enopyranosyl) generated from alginate degradation. (d) The key residues involved in alginate binding in the B1 domain. (e) An overview of conserved residues involved in alginate binding of the B1 domain and its homologues. (f) The binding ability of B1 and its mutants toward alginate gel beads. The graphs show data from triplicate experiments (mean ± SD).

**
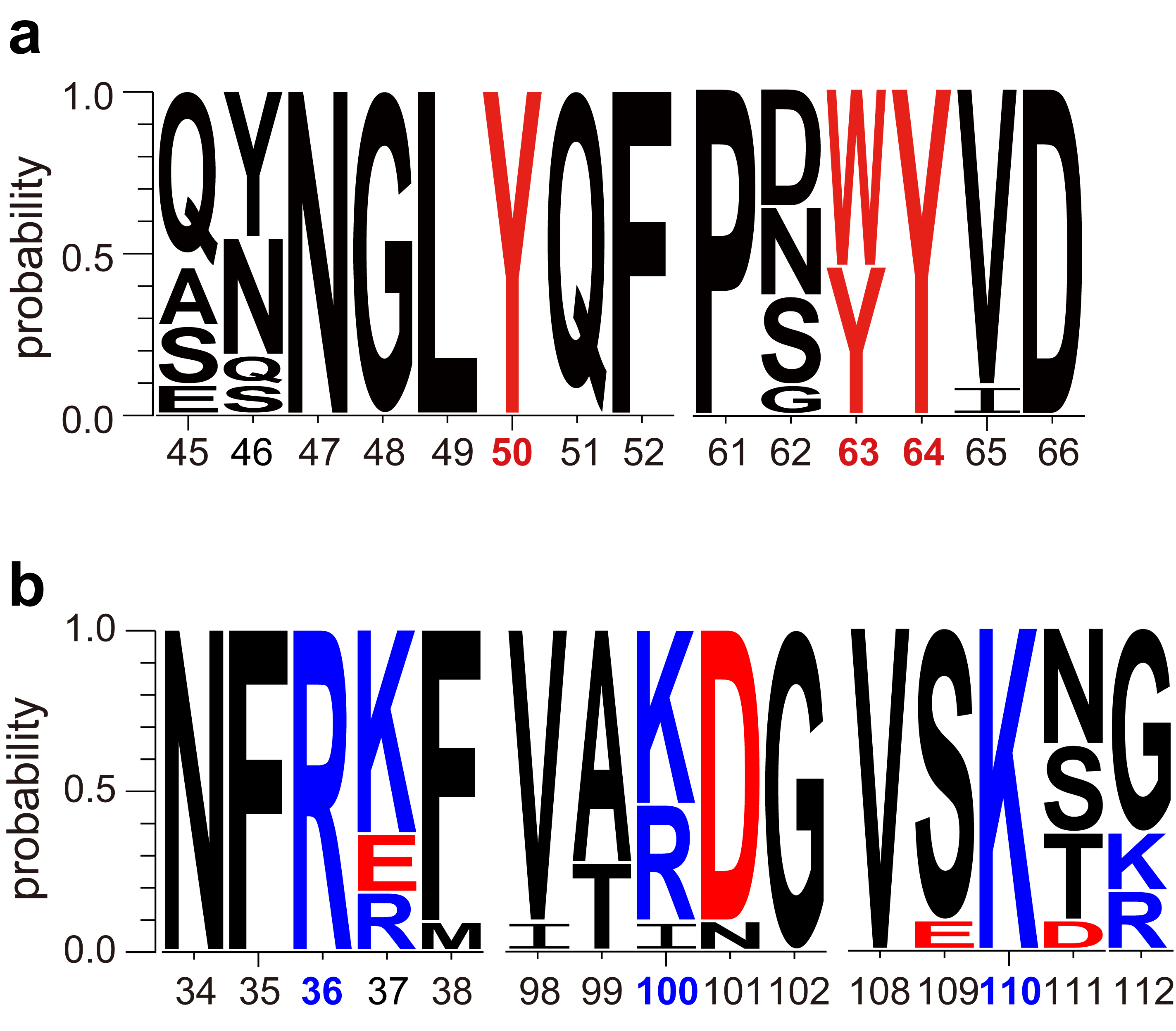
**

**Figure S6. An overview of conserved residues involved in insoluble cellulose (a) and alginate (b) binding of the UKD domain and its homologues.**


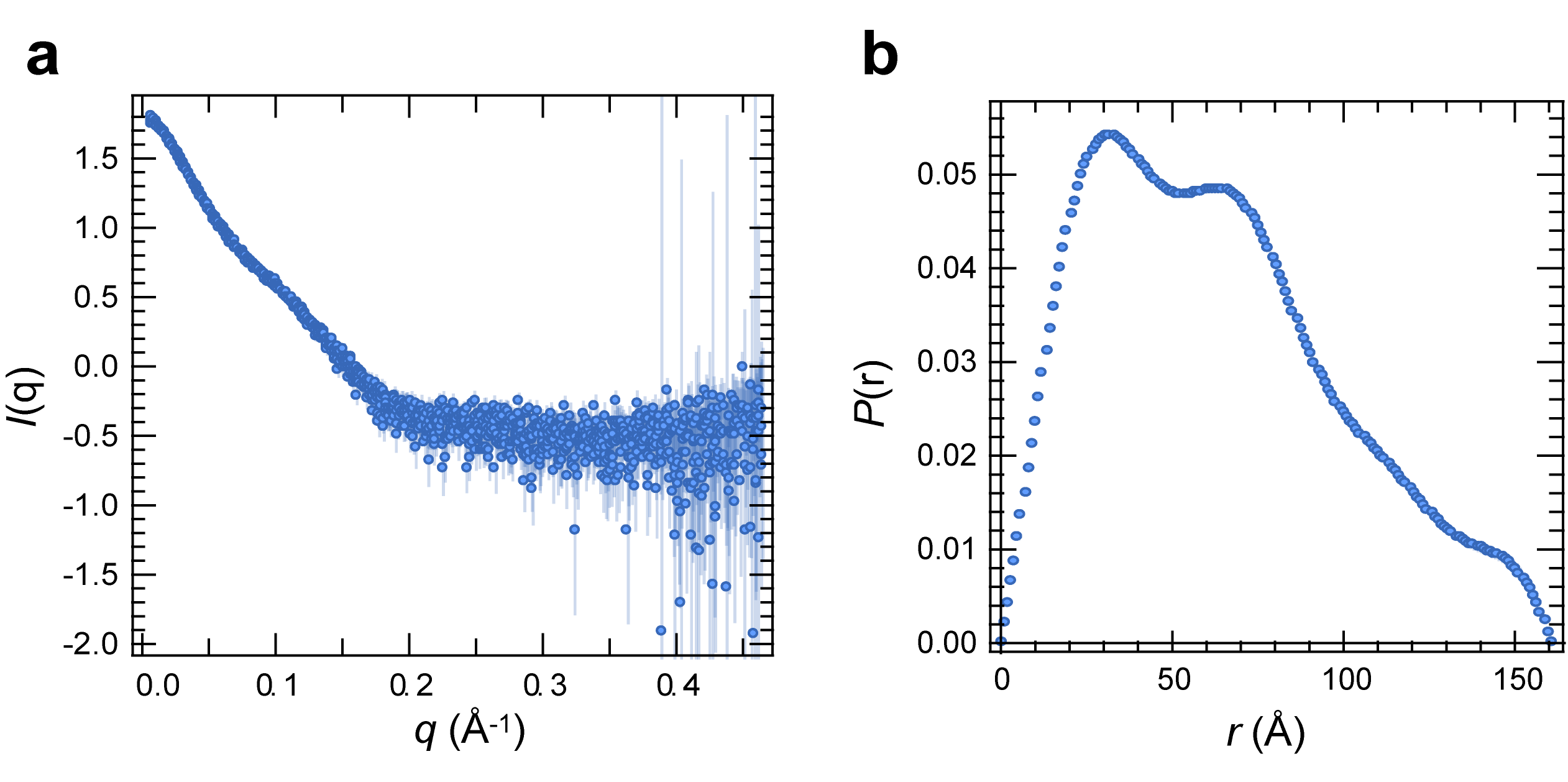


**Figure S7. SAXS data of recCelAly.** (a) Experimental scattering profile for recCelAly. (b) The *P*(*r*) distribution for recCelAly. The figures were generated using PRIMUS in the ATSAS software package.


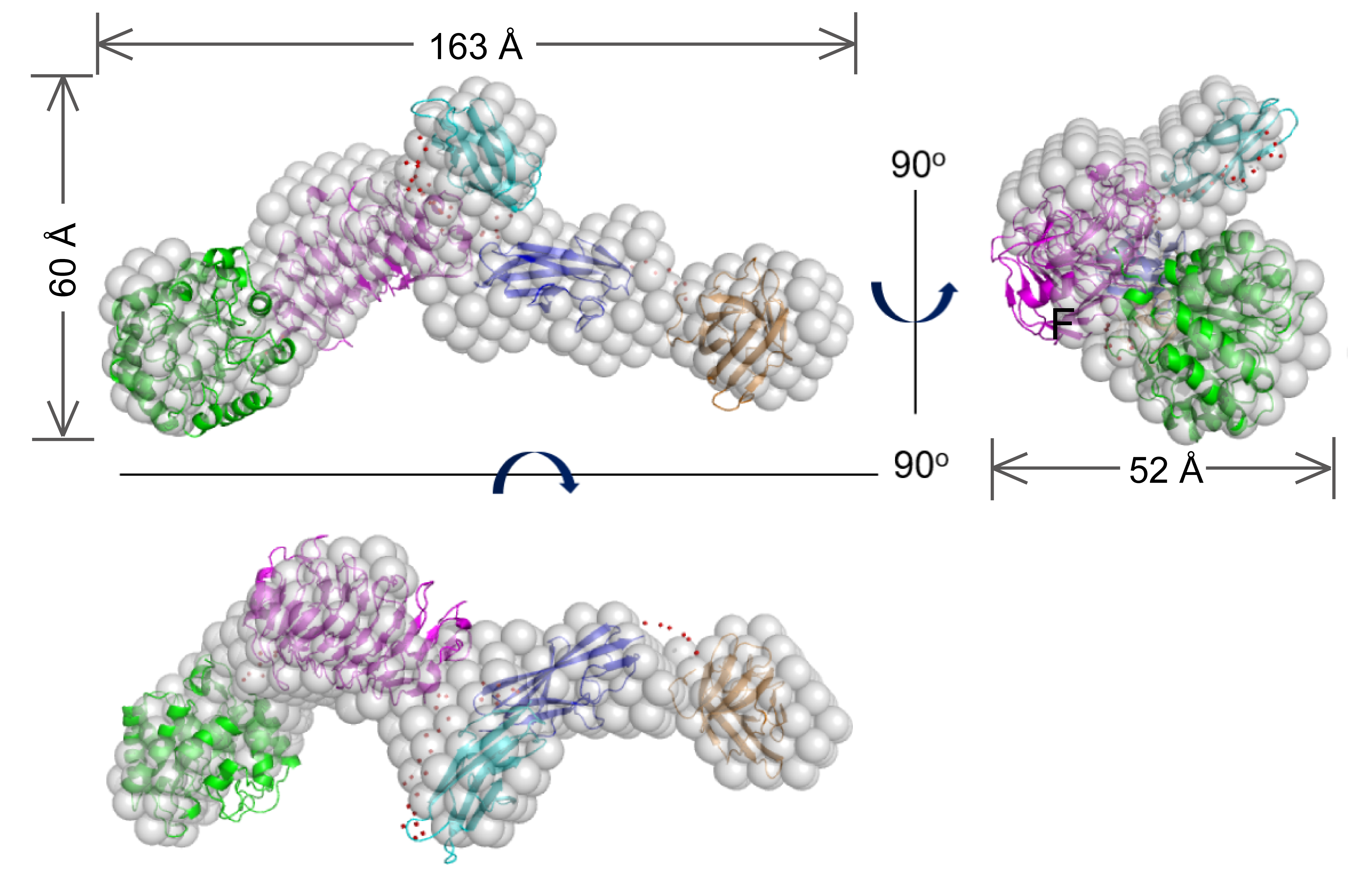


**Figure S8. The overall structure of recCelAly.** The beads model is represented as grey spheres. The rigid body model is drawn as a cartoon representation and different colors represent different domains of CelAly. The poly-glycine linker added by CORAL is shown in red.

**
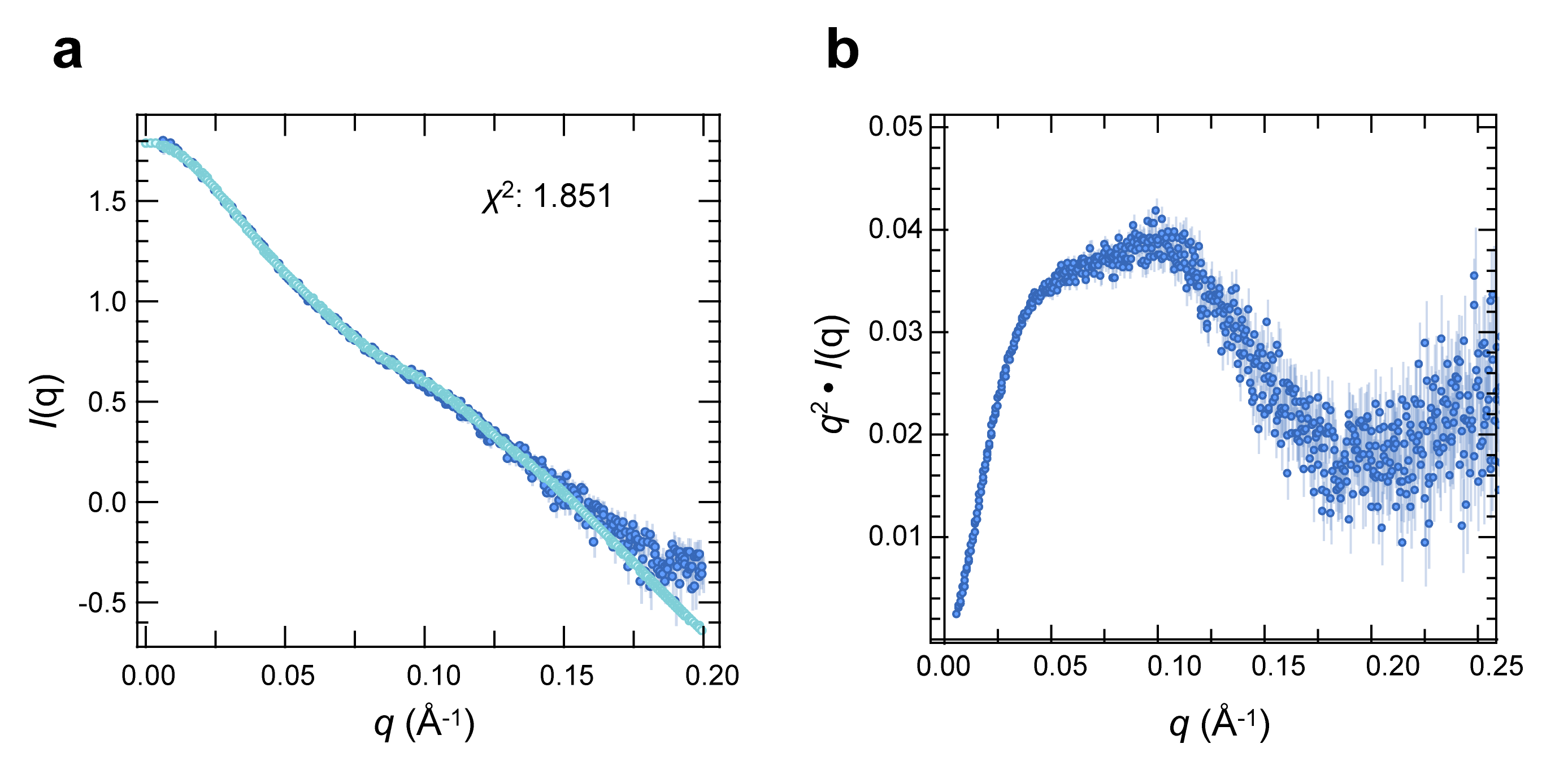
**

**Figure S9.** **The theoretical scattering curve of the rigid body model fitted to the experimental curve (a) and the Kratky plot (b) for recCelAly.** The figures were generated using PRIMUS in the ATSAS software package.

**Table S1.** **Primers for gene cloning in this study*^a^*.**

| **Gene product** | **Primer** | **Sequence (5’ to 3’)** |
| --- | --- | --- |
| recCelAly | recCelAly-F | AAGAAGGAGATATACATATGTGCGCACAGACAGTTGTGCA |
|  | recCelAly-R | TGGTGGTGGTGGTGCTCGAGTTTTGCGGCAGTCGTTTTCA |
| TM1 | TM1-F | AAGAAGGAGATATACATATGAACATTGTAAATCCTGATCC |
|  | TM1-R | TGGTGGTGGTGGTGCTCGAGTTTTGCGGCAGTCGTTTTCA |
| TM2 | TM2-F | AAGAAGGAGATATACATATGTGCGCACAGACAGTTGTGCA |
|  | TM2-R | TGGTGGTGGTGGTGCTCGAGGTTTCGGATACAAGCTACCG |
| TM3 | TM3-F | AAGAAGGAGATATACATATGAACATTGTAAATCCTGATCC |
|  | TM3-R | TGGTGGTGGTGGTGCTCGAGTCCTGAACATGGGACTATGT |
| TM4 | TM4-F | AAGAAGGAGATATACATATGGGTGGTAATCAAGATCCGAC |
|  | TM4-R | TGGTGGTGGTGGTGCTCGAGACCAGAGACTGTAACATTTA |
| TM5 | TM5-F | AAGAAGGAGATATACATATGAGTACTGGCGGAGGTAACAT |
|  | TM5-R | TGGTGGTGGTGGTGCTCGAGCGTACTGCTACCACCAGTAT |
| TM6 | TM6-F | AAGAAGGAGATATACATATGTGTAGTTTTGGAACTCCGAC |
|  | TM6-R | TGGTGGTGGTGGTGCTCGAGTTTTGCGGCAGTCGTTTTCA |

*^a^* Restriction enzyme sites used for cloning are underlined.

**Table S2. Diffraction data and refinement statistics of the B1 and UKD domain.**

| **Parameter** | **B1** | **UKD** |
| --- | --- | --- |
| **Data collection** |  |  |
| Beamline | SSRF BL02U1 | SSRF BL02U1 |
| Space group | P 21 21 21 | P 4 3 2 |
| Unit cell*^a^* |  |  |
| *a* (Å) | 30.85 | 142.86 |
| *b* (Å) | 41.69 | 142.86 |
| *c* (Å) | 53.95 | 142.86 |
| *α* (°) | 90.00 | 90.00 |
| *β* (°) | 90.00 | 90.00 |
| γ (°) | 90.00 | 90.00 |
| Wavelength (Å) | 0.9792 | 0.9792 |
| Resolution (Å) | 32.99-1.90 (1.94-1.90) | 82.48-2.70 (2.83-2.70) |
| Multiplicity | 11.6 (8.9) | 69.4 (58.5) |
| Completeness (%) | 98.14 (96.88) | 97.91 (100.00) |
| *R*_merge_*^b^* | 0.170 (0.659) | 0.078 (0.828) |
| *I/σ* | 8.6 (2.4) | 49.3 (7.1) |
| **Refinement statistics** |  |  |
| Resolution (Å) | 26.78-1.90 (1.97-1.90) | 31.94-2.70 (2.80-2.70) |
| *R*_work_ (%) | 20.59 (29.88) | 21.79 (36.37) |
| *R*_free_ (%) | 24.00 (35.17) | 27.80 (40.49) |
| B-factor (Å^2^) |  |  |
| Protein | 30.87 | 62.62 |
| Solvent | 32.99 | 59.29 |
| Ligands | 30.00 |  |
| RMSD from ideal geometry |  |  |
| Length (Å) | 0.007 | 0.012 |
| Angels (°) | 1.05 | 1.29 |
| Ramachandran plot (%)*^c^* |  |  |
| Favored | 97.83 | 95.97 |
| Allowed | 2.17 | 4.03 |
| **PDB codes** | **9KVU** | **9KVN** |

*^a^* Numbers in parentheses refer to data in the highest resolution shell.

*^b^* R_merge_ = Σ_hkl_Σ_i_|*I*(*hkl*)_i_ - <*I*(*hkl*)>|/Σ_hkl_Σ_i_ < *I*(*hkl*)_i_>.

**Table S3. SAXS parameters of recCelAly.**

| **Parameters** | **recCelAly** |
| --- | --- |
| **Data Collection** |  |
| Beamline | SSRF (Shanghai, China) BL19U2 |
| Wavelength (Å) | 1.54 |
| Detector Pilatus | Pilatus 1M |
| *q* range (Å^-1^) | 0.007-0.47 |
| Exposure time (s) | 1 s for 20 frames |
| Protein concentration (mg/ml) | 0.5 to 5 mg/mL |
| Temperature (^o^C) | 10 |
| **Structural Parameters** |  |
| *I*(0) (cm^-1^) from Guinier fit | 50.64 |
| *R*_g_ (Å) from Guinier fit | 62.07 |
| *I*(0) (cm^-1^) from *P*(*r*) | 50.94 |
| *R*_g_ (Å) from *P*(*r*) | 62.08 |
| *D*_max_ (Å) from *P*(*r*) | 160.9 |
| **Modeling** |  |
| DAMMIN χ^2^ | 1.206 |
| DAMMIN Ensemble Resolution | 50 ± 4 |
| DAMMIN NSD | 0.793 ± 0.051 |
| **Software Employed** |  |
| Primary data Processing | RAW |
| *P*(*r*) | GNOM |
| *Ab initio* shape analysis | DAMMIF |
| Rigid body modeling | CORAL |
| SAXS Profile computation | CRYSOL |
| Molecular Visualization | PyMOL |

**Table S4. The substrates used in this study.**

|  | **Substrate** | **Description** |
| --- | --- | --- |
| **Glucan** | CMC | Substrate for endo-β-1,4-glucanase |
|  | HEC | Substrate for endo-β-1,4-glucanase |
|  | PASC | Substrate for endo-β-1,4-glucanase |
|  | Avicel | Substrate for exo-β-1,4-glucanase |
|  | Barley β-D-glucan | Substrate for β-1,3-1,4-glucanase |
|  | Laminarin | Substrate for β-1,3/1,6-glucanase |
| **Xylan** | β-1,4-xylan | Substrate for endo-β-1,4-xylanase |
|  | β-1,3/1,4-xylan | Substrate for endo-β-1,3-1,4-xylanase |
| **Alginate** | Sodium alginate | Substrate for alginate lyase |
|  | PMG | Substrate for MG-specific alginate lyase |
|  | PM | Substrate for PM-specific alginate lyase |
|  | PG | Substrate for PG-specific alginate lyase |
| **Others** | Heparan sulfate | Substrate for heparin lyase |
|  | Chondroitin sulfate | Substrate for chondroitin lyase |
|  | Hyaluronic acid | Substrate for hyaluronate lyase |
|  | Ulvan | Substrate for ulvan lyase |

CMC, carboxymethyl cellulose. HEC, hydroxyethyl cellulose. PASC, phosphoric acid swollen cellulose. PMG, heteropolymers consisting of M and G alternately. PM, polymannuronate. PG, polyguluronate.

T**able S5. The enzyme activity of recCelAly's mutants with key catalytic amino acid residue mutations.**

| **Enzyme activity type** | **Enzyme** | **Relative activities (%)** |
| --- | --- | --- |
| Cellulase activity | recCelAly | 100 |
|  | E173A | 0 |
|  | E259A | 0.072 ± 0.009 |
| Alginate lyase activity | recCelAly | 100 |
|  | Y501A | 0 |
|  | K538A | 0.030 ± 0.001 |

**Table S6. The putative BACWP-degrading enzymes** **from marine sources in the CAZy and NCBI databases.**

| **Type** | **Accession No.** | **Strain** | **Source** |
| --- | --- | --- | --- |
| GH5+PL31 | WP_407141409.1 **(CelAly)** * | *Aquimarina* sp. 2-A2 | Sediment in the Southwest Indian Ocean |
|  | WP_456516552.1 **(CELALY’)** | *Aquimarina* sp. 433 | Seawater in Qingdao, China |
|  | ADY28795.1*^a^* | *Cellulophaga lytica* DSM 7489 | Beach mud |
|  | APU09708.1*^a^* | *Cellulophaga lytica* DAU203 | Marine sediment in Busan, South Korea |
|  | APY12487.1*^a^* | *Seonamhaeicola* sp. S2-3 | Seawater in Jeju island, South Korea |
|  | AUC74935.1*^a^* | *Olleya* sp. Bg11-27 | Sea ice in near Point Barrow, Alaska |
|  | AXO78975.1*^a^* | *Olleya aquimaris* DAU311 | Marine sediment in Goraebul beach, South Korea |
|  | AXT51607.1*^a^* | *Aquimarina* sp. BL5 | Seaweed *Delisea pulchra* on the coast of Sydney |
|  | AXT57650.1*^a^* | *Aquimarina* sp. AD1 | Seaweed *Delisea pulchra* on the coast of Sydney |
|  | AXT58949.1*^a^* | *Aquimarina* sp. AD10 | Seaweed *Delisea pulchra* on the coast of Sydney |
|  | QCE43156.1*^a^* | *Psychroserpens* sp. NJDZ02 | Macroalgae in Antarctic |
|  | QXP60845.1*^a^* | *Olleya* sp. HaHaR_3_96 | Surface seawater in North Sea |
|  | SNR15156.1*^a^* | *Tenacibaculum jejuense* KCTC 22618 | Coastal seawater in Jeju Island, South Korea |
|  | WZL62533.1*^a^* | *Aquimarina* sp. U1-2 | Ocean in Shantou, China |
|  | MAQ75930.1 | *Aquimarina* sp. NAT579 | North Atlantic Ocean |
|  | WP_051336141.1 | *Aquimarina latercula* DSM 2041 | Seawater aquarium outflow |
|  | WP_066314690.1 | *Aquimarina aggregata* RZW4-3-2 | Coastal surface seawater of the Yellow Sea |
|  | WP_073315802.1 | *Aquimarina spongiae* DSM 22623 | Marine sponge *Halichondria oshoro* on the coast of Jeju Island, South Korea |
|  | WP_082395983.1 | *Olleya* sp. ITB9 | Water in Tokyo Bay, Japan |
|  | WP_089118852.1 | *Cellulophaga lytica* CL8139 | Red anemone (*Actinia equine*) surface |
|  | WP_091411656.1 | *Aquimarina amphilecti* DSM 25232 | Marine sponge in Lough Hyne, Ireland |
|  | WP_105048247.1 | *Polaribacter butkevichii* KCTC 12100 | Seawater in Japan |
|  | WP_158837275.1 | *Polaribacter* sp. L3A8 | Beach brown algae in Arctic Ocean |
|  | WP_159092196.1 | *Aquimarina* sp. Aq107 | Marine sponge in Algarve, Gale Alta |
|  | WP_168036730.1 | *Neolewinella antarctica* DSM 105096 | Seawater in Antarctica |
|  | WP_169684745.1 | *Marinigracilibium pacificum* KN852 | Seawater |
|  | WP_201916987.1 | *Aquimarina mytili* JCM 17454 | Mussel *Mytilus coruscus* in South Korea |
|  | WP_203001601.1 | *Olleya sediminilitoris* YSTF-M6 | Tidal flat sediment in Incheon, South Korea |
|  | WP_211062010.1 | *Aquimarina* sp. MMG015 | Tubeworm in Sunset Cliffs, San Diego |
|  | WP_211073589.1 | *Aquimarina* sp. MMG016 | Purple seaweed in Sunset Cliffs, San Diego |
|  | WP_219011040.1 | *Aquimarina litoralis* CCMR20 | Mussismilia braziliensis coral |
|  | WP_238749638.1 | *Neolewinella maritima* CECT 8419 | Seawater in South Korea |
|  | WP_248334086.1 | *Aquimarina acroporae* D1M17 | Seawater in China |
|  | WP_281988247.1 | *Aquimarina aggregata* 2-322 | Marine macroalgae in Weihai, China |
|  | WP_298313963.1 | uncultured *Aquimarina* sp. 490 | Marine macroalgae in Weihai, China |
|  | WP_298541871.1 | uncultured *Aquimarina* sp. 4R5-1-G | Marine macroalgae in Weihai, China |
|  | WP_299188565.1 | uncultured *Aquimarina* sp. X6 | Marine macroalgae in Weihai, China |
|  | WP_299223612.1 | uncultured *Aquimarina* sp. 3-916-3Z | Marine macroalgae in Weihai, China |
|  | WP_299245976.1 | uncultured *Aquimarina* sp. 2-32 | Marine macroalgae in Weihai, China |
|  | WP_299258446.1 | uncultured *Aquimarina* sp. 3-1138-G | Marine macroalgae in Weihai, China |
|  | WP_299313152.1 | uncultured *Aquimarina* sp. 3-716 | Marine macroalgae in Weihai, China |
|  | WP_299432953.1 | uncultured *Aquimarina* sp. 2-563A-J | Marine macroalgae in Weihai, China |
|  | WP_299604102.1 | uncultured *Aquimarina* sp. 550 | Marine macroalgae in Weihai, China |
|  | WP_299680978.1 | uncultured *Tenacibaculum* sp. 4-641-3Z | Marine macroalgae in Weihai, China |
|  | WP_299894584.1 | uncultured *Aquimarina* sp. 740 | Marine macroalgae in Weihai, China |
|  | WP_303545323.1 | *Cellulophaga* sp. G2R07 | Canoe Beach, Nahant, MA, USA |
|  | WP_324179839.1 | *Aquimarina gracilis* JCM 17453 | Mussel *Mytilus coruscus* from Gwangyang Bay |
|  | WP_343911371.1 | *Aquimarina litoralis* JCM 15974 | Seawater off the coast of Jeju Island |
|  | WP_344926093.1 | *Aquimarina addita* JCM 17106  *JCM 17106* | Seawater from Jeju Island |
|  | WP_348129482.1 | *Cellulophaga* sp. AQ.63.F.D4_12_70 | *Macrocystis pyrifera* in Arroyo Quemado, California |
|  | WP_349664404.1 | *Cellulophaga* *lytica* FXS1 | Algae in Portugal  [Portugal](https://www.ncbi.nlm.nih.gov/biosample?term=%22geo_loc_name=Portugal%22%5battr%5d)  [Portugal](https://www.ncbi.nlm.nih.gov/biosample?term=%22geo_loc_name=Portugal%22%5battr%5d) |
|  | WP_367583268.1 | *Aquimarina* sp. 2304DJ70-9 | Sponge *Penares incrustans* in Jeju-do, South Korea |
|  | WP_367589232.1 | *Aquimarina* sp. 2201CG1-2-11 | Sponge *Discodermia calyx* in Chagwido-island, South Korea  [South Korea: Chagwido-island](https://www.ncbi.nlm.nih.gov/biosample?term=%22geo_loc_name=South%20Korea:%20Chagwido-island,%20Jeju-do%22%5battr%5d)  [South Korea: Chagwido-island](https://www.ncbi.nlm.nih.gov/biosample?term=%22geo_loc_name=South%20Korea:%20Chagwido-island,%20Jeju-do%22%5battr%5d) |
|  | WP_378181209.1 | *Aquimarina* sp. SS2-1 | Marine algae in Gunsan, South Korea |
|  | WP_378187021.1 | *Aquimarina* sp. W85 | Marine algae in Taean, South Korea |
|  | WP_386406786.1 | *Sungkyunkwania multivorans* CCUG 62952 | Seawater from a seaweed farm on the South Sea in Korea |
|  | WP_405208480.1 | *Aquimarina* sp. LLG6339-5 | *Cystoseira* sp. from Corsica near Negru, France |
|  | WP_452056711.1 | *Aquimarina* sp. M1 | Ulva in Muan Bay, South Korea |
| PL14+GH9 | QPH54235.1 **(Enzyme A)** *^a^*^,^* | *Pontivivens ytuae* MT2928 | Deep sea sediment in Mariana Trench |
|  | MDP4625328.1 | Akkermansiaceae bacterium MAG-316 | Seawater in Kalmar Sound, Sweden |
|  | MFK7910692.1 | Akkermansiaceae bacterium SGMAG-39 | *Zostera marina* in Bodega Bay, CA, USA |
|  | WP_200283071.1 | *Haloferula rosea* KCTC 22201 | Marine sponge in Nagate Cape, Sado Island, Niigata, Japan |
|  | WP_308950265.1 | *Thalassobacterium maritimum* | Sediment from intertidal zone in Weihai, China |
|  | WP_308986336.1 | *Thalassobacterium sedimentorum* | Sediment from intertidal zone in Weihai, China |
|  | WP_319833968.1 | *Coraliomargarita algicola* J2-16 | Marine algae in South Korea |
|  | WP_338689921.1 | *Haloferula helveola* | Seawater in estuary of Urauchi River, Japan |
|  | WP_204414665.1 | *Actibacterium* sp. 188UL27-1 | *Chaetomorpha moniligera* in Ulleung Island, South Korea |
|  | MEL6838219.1 | Pseudomonadota bacterium | Plastic particle in Atlantic Ocean |
| PL7+GH16+PL6 | WBB59582.1 **(Enzyme B)** *^a^*^,^* | *Streptomyces* sp. WMMC500 | *Ecteinascidia turbinate* in Ramrod Key, Florida, USA |
|  | AUH45140.1*^a^* | *Streptomyces* sp. CMB-StM0423 | Beach sand in Heron Island, Queensland, Australia |
|  | MFW6724244.1 | *Streptomyces* sp. MAR4 CNY-716 | Off the coast of Southern California |
|  | WP_047015698.1 | *Streptomyces* sp. CNQ-509 | Marine sediment in 1 mile off Scripps Pier, CA, USA |
|  | WP_234380489.1 | *Streptomyces* sp. CMB-StM0423 | Beach sand in Heron Island, Queensland, Australia |
|  | WP_420303927.1 | *Streptomyces* sp. MAR4 CNX-425  *Streptomyces sp. MAR4 CNX-425* | Off the coast of Southern California |
| PL7+GH16 | ARQ72427.1 **(Enzyme D)** *^a^*^,^* | *Streptomyces marincola* SCSIO 03032 | Deep marine sediment in Bay of Bengal, Indian Ocean |
|  | UCM88509.1*^a^* | *Streptomyces marincola* SCSIO 64649 | Coral in Sanya, Hainan, China |
|  | UED88401.1*^a^* | *Streptomyces* sp. MA3_2.13 | Deep-sea sediment in Atlantic Ocean |
|  | UNS95275.1*^a^* | *Streptomyces* sp. DSD3025 | Marine sediment Philippines in Tubbataha Reefs National Park, Philippines |
|  | MBO8197352.1 | *Streptomyces smyrnaeus* DSM 42105 | Saltern sediment in Turkey |
|  | RCG15713.1 | *Streptomyces diacarni* LHW51701 | Marine sponge in Xisha, China |
|  | WP_053171341.1 | *Streptomyces* sp. SBT349 | *Sarcotragus spinosulus* in 5-7 m offshore Pollonia, Greece |
|  | WP_120674164.1 | *Streptomyces hoynatensis* KCTC 29097 | Marine sediment in black sea coast, Turkey |
|  | WP_147255653.1 | *Streptomyces* sp. PT12 | Marine sponge in Pulau Tioman Malaysia |
|  | WP_228079783.1 | *Streptomyces profundus* | Deep-sea sediment in Atlantic Ocean |
|  | WP_245224812.1 | *Streptomyces smyrnaeus* DSM 42105 | Saltern sediment in Turkey |
|  | WP_245980026.1 | *Streptomyces diacarni* LHW51701 | Marine sponge in Xisha, China |
|  | WP_255308168.1 | *Streptomyces marincola* SCSIO 03032 | Sediment in Bay of Bengal, Indian Ocean |
|  | WP_311597602.1 | *Streptomyces millisiae* DSM 44918 | Marine sediment in Guam, USA |
|  | WP_311704727.1 | *Streptomyces litchfieldiae* DSM 44938 | Marine sediment in Bahamas |

*^a^* The protein sequences were identified by searching the CAZy database.

* The protein sequences that have been heterologously expressed for activity determination.
